# Using intervention mapping to develop a theory-driven, group-based complex intervention to support self-management of osteoarthritis and low back pain (SOLAS)

**DOI:** 10.1186/s13012-016-0418-2

**Published:** 2016-04-26

**Authors:** Deirdre A. Hurley, Laura Currie Murphy, David Hayes, Amanda M. Hall, Elaine Toomey, Suzanne M. McDonough, Chris Lonsdale, Nicola E. Walsh, Suzanne Guerin, James Matthews

**Affiliations:** 1 UCD School of Public Health, Physiotherapy and Sports Science, University College Dublin, Dublin 4, Ireland; 2 Breast-Predict-Collaborative Cancer Research Centre, Pharmacology and Therapeutics, Trinity College Dublin, St James’s Hospital, Dublin 8, Ireland; 3 UCD School of Psychology, University College Dublin, Dublin 4, Ireland; 4 The George Institute for Global Health, Oxford Martin School, Oxford University, Oxford, UK; 5 Institute of Nursing and Health Research, Jordanstown Campus, Ulster University, Antrim, BT37 0QB UK; 6 Institute for Positive Psychology and Education, Faculty of Health Sciences, Australian Catholic University, 25A Barker Road, Strathfield, NSW 2135 Australia; 7 Faculty of Health and Applied Sciences, Glenside Campus, University of the West of England, Bristol, BS16 1DD UK

**Keywords:** Intervention mapping, Complex group intervention, Behaviour change intervention, Self-management, Physical activity, Mixed methods, Physiotherapists, Patient-public involvement, Osteoarthritis, Low back pain

## Abstract

**Background:**

The Medical Research Council framework provides a useful general approach to designing and evaluating complex interventions, but does not provide detailed guidance on how to do this and there is little evidence of how this framework is applied in practice. This study describes the use of intervention mapping (IM) in the design of a theory-driven, group-based complex intervention to support self-management (SM) of patients with osteoarthritis (OA) and chronic low back pain (CLBP) in Ireland’s primary care health system.

**Methods:**

The six steps of the IM protocol were systematically applied to develop the self-management of osteoarthritis and low back pain through activity and skills (SOLAS) intervention through adaptation of the Facilitating Activity and Self-management in Arthritis (FASA) intervention. A needs assessment including literature reviews, interviews with patients and physiotherapists and resource evaluation was completed to identify the programme goals, determinants of SM behaviour, consolidated definition of SM and required adaptations to FASA to meet health service and patient needs and the evidence. The resultant SOLAS intervention behavioural outcomes, performance and change objectives were specified and practical application methods selected, followed by organised programme, adoption, implementation and evaluation plans underpinned by behaviour change theory.

**Results:**

The SOLAS intervention consists of six weekly sessions of 90-min education and exercise designed to increase participants’ physical activity level and use of evidence-based SM strategies (i.e. pain self-management, pain coping, healthy eating for weight management and specific exercise) through targeting of individual determinants of SM behaviour (knowledge, skills, self-efficacy, fear, catastrophizing, motivation, behavioural regulation), delivered by a trained physiotherapist to groups of up to eight individuals using a needs supportive interpersonal style based on self-determination theory. Strategies to support SOLAS intervention adoption and implementation included a consensus building workshop with physiotherapy stakeholders, development of a physiotherapist training programme and a pilot trial with physiotherapist and patient feedback.

**Conclusions:**

The SOLAS intervention is currently being evaluated in a cluster randomised controlled feasibility trial. IM is a time-intensive collaborative process, but the range of methods and resultant high level of transparency is invaluable and allows replication by future complex intervention and trial developers.

**Electronic supplementary material:**

The online version of this article (doi:10.1186/s13012-016-0418-2) contains supplementary material, which is available to authorized users.

## Background

Highly prevalent chronic musculoskeletal pain conditions, such as osteoarthritis (OA) and chronic low back pain (CLBP), place substantial burdens on individuals, health systems, and economies through their profound impact on physical function, psychosocial well-being, quality of life and productivity [1–3]. Clinical guidelines endorse patient education about the underlying chronic condition and support for self-management (SM) behaviours, including physical activity [4–7], with SM programmes being championed in many health systems [8–10] internationally, but there has been minimal implementation in primary care in Ireland [11]. Contributing factors include variability in how SM is defined in the literature [12], the small effects for interventions in OA [13], the limited evidence base for effective interventions in CLBP [14] management and the diverse case mix of patients in primary care, which limits the time and expertise [15, 16] of physiotherapists tasked with developing such programmes [17]. Furthermore, the variable quality of Ireland’s primary care health system infrastructure and staffing levels present further barriers [11], which taken together have contributed to a ‘second translational gap’ [18].

A systematic review of SM interventions for a range of chronic musculoskeletal pain conditions found that short (<8 weeks), healthcare professional-delivered, group interventions showed some positive effects, but further research of their effectiveness and cost-effectiveness was warranted [19]. The successful implementation of a standardised, evidence-based clinical and cost-effective group programme to support SM for patients with chronic musculoskeletal pain is a key priority for primary care physiotherapy in Ireland [9]; however, a potential intervention must first be demonstrated to be credible, feasible and implementable within this challenging health service context prior to widespread adoption.

Complex interventions, for example, those designed to improve health outcomes by changing SM behaviour, contain several interacting components, as well as variability within the range of possible outcomes and number of behaviours required by those delivering and receiving the intervention [20]. They typically include behavioural support to improve adherence to the desired behaviour and may target both modifications in healthcare provider behaviour relating to how they interact with patients in delivering the intervention and patient behaviour in adopting it. Moreover, the causal chain linking a behavioural support intervention to health outcomes is complex and requires a relevant theoretical model to understand its mechanisms of action [21–23]. This is further challenged by the demands associated with standardising the design and delivery of the intervention, sensitivity to local context, the organisational and logistical difficulties of applying standard experimental methods and the length and complexity of the causal chains [20]. Indeed, it has been acknowledged that ensuring strict standardisation may be inappropriate and the intervention may work better if a specified degree of adaptation to local settings is allowed [20]. Nonetheless, a change in usual clinical practice is often required to ensure successful implementation, notwithstanding the additional complexity of delivering a group intervention [24].

The Medical Research Council (MRC) updated guidelines recommend an iterative, cyclical phased approach to intervention development and evaluation [20, 25–27], noting that ‘too strong an emphasis on the main evaluation to the neglect of adequate development and piloting or consideration of the practical issues of implementation will result in weaker interventions that are harder to evaluate, less likely to be implemented and less likely to be worth implementing’ [20]. Concern for implementation should begin in the design phase through consideration of the barriers and enablers to successful implementation and engagement of key stakeholders through involvement in the design and feasibility processes. The MRC framework provides a useful general approach to designing and evaluating complex interventions, but it does not provide detailed guidance on how to do this [28]. While the evaluation phase is widely reported with improving transparency [29], there are few published examples of how the wider aspects of this framework are applied in practice [30, 31]. Intervention mapping (IM) provides a logical process for intervention development, implementation and evaluation [32] that fulfils the MRC framework criteria and has been previously used to develop [33] and adapt evidence-based SM programmes for other settings [34]. The primary aims of this study were to use the IM process to develop a complex group-based SM intervention (SOLAS: self-management of osteoarthritis and low back pain through activity and skills) for Ireland’s primary care physiotherapy service through adaptation of an existing evidence-based programme (Facilitating Activity and Self-management in Arthritis (FASA) [35]) which would serve as a prototype and to address factors related to its implementation in a planned feasibility trial [36] set in the publicly-funded Health Service Executive Primary Community and Continuing Care (PCCC) physiotherapy services of Dublin, Kildare and Wicklow on the east coast of Ireland serving a population of 1.6 million [37].

## Methods

**Fig. 1:**
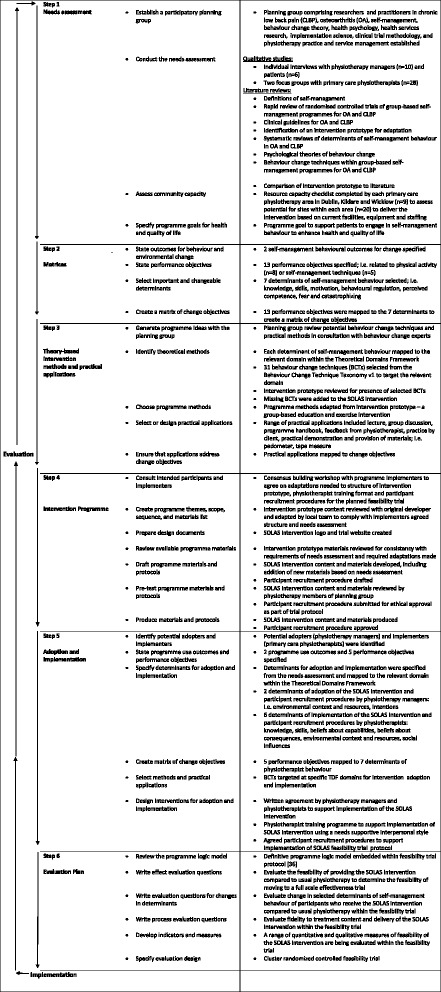
Intervention mapping process, Bartholomew et al. [32]

IM is a six-step process with each step consisting of several tasks which once completed inform the next step as detailed in Bartholomew et al. [32] and in Fig. 1.

- Step one: needs assessment
  - The aim of step 1 was to develop programme goals for the intervention related to health and quality of life based on a detailed multi-method assessment of the needs of the PCCC physiotherapy service providers and patients and the literature regarding SM for chronic musculoskeletal pain to establish how an intervention could be designed to meet these needs.
  - Semi-structured interviews
    - Individual semi-structured, qualitative interviews were conducted with all consenting physiotherapy managers (*n* = 10) in the catchment area of the feasibility trial and a sample of consenting patients with CLBP and/or spinal OA (*n* = 6) who had recently participated in a group-based physiotherapy programme to understand their needs in relation to a SM intervention. Both studies were approved by the UCD Human Research Ethics Committee-Sciences (Ref no: LS-E-13-103-Hurley-Osing; Ref no: LS-13-25-Toomey-Hurley-Osing). Deductive thematic analysis based on Braun and Clarke’s method [38] was conducted on the data using the Theoretical Domains Framework (TDF) [39]. The TDF is a validated integrative framework that synthesised key theoretical constructs from 33 behaviour change theories into 14 domains that supports the identification and selection of relevant determinants of behaviour for targeting within interventions. An additional file provides details of the interview topic guides and coding frames (see Additional file 1).
  - Literature reviews
    - A thematic analysis of chronic disease SM definitions was conducted to reach a consolidated definition. This process is shown in detail in an additional file (see Additional file 2). This definition was then applied to a rapid review of the effectiveness of physiotherapy delivered group-based SM programmes for OA and CLBP, which was lacking in the literature. An intervention prototype was identified for further adaptation based on its evidence base, similarities in health service context and relevance to the target populations. The most recent international clinical guideline recommendations relating to programme content and SM behaviour for OA and CLBP were reviewed. The behavioural determinants of outcomes of SM interventions identified in recent systematic reviews within the target populations, general behaviour change theories, and behaviour change theories and techniques (BCTs) reported in systematic reviews of SM interventions and our rapid review [40] were reviewed for their relevance to targeting and supporting adherence to SM behaviours [41]. The intervention prototype was then compared to the literature to identify necessary adaptations for SOLAS.

**Fig. 2:**
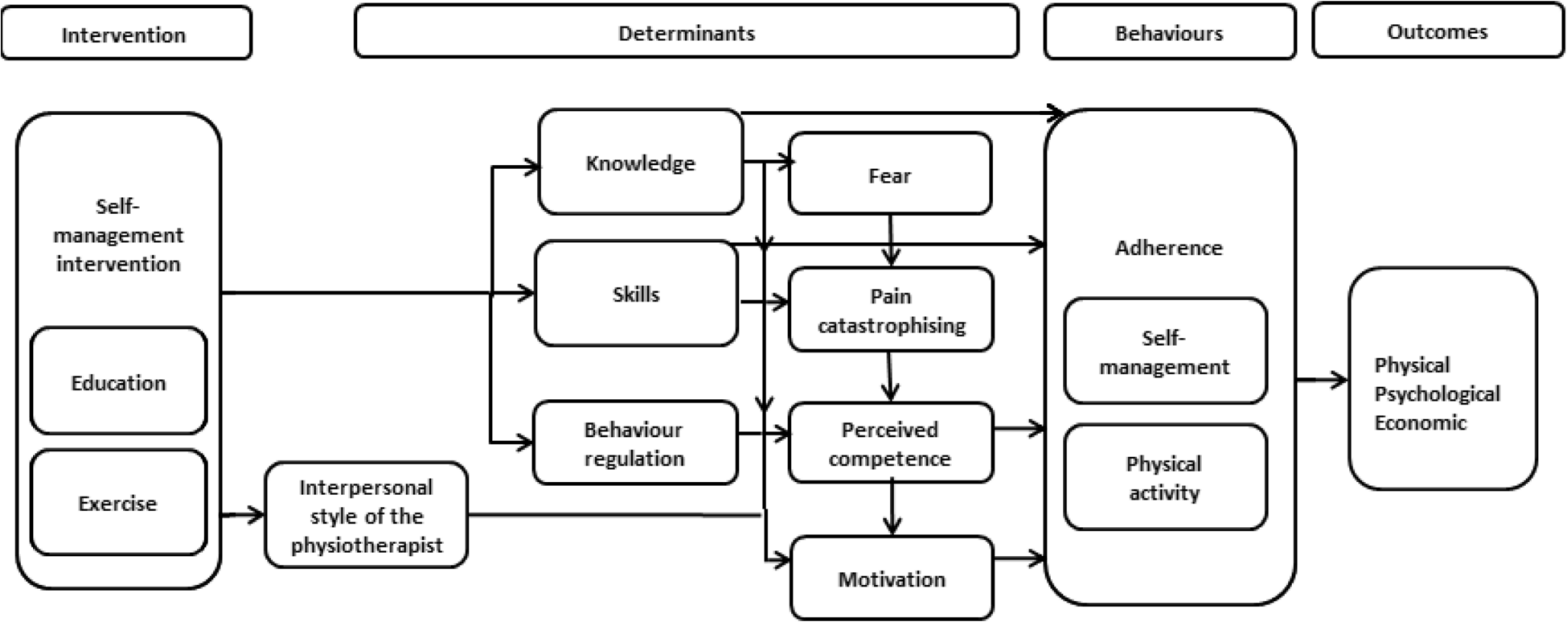
Theoretical framework of behaviour change for SOLAS intervention
  - Focus groups
    - Two focus groups with purposively selected consenting physiotherapists (*n* = 28) working in the catchment area were conducted to explore the feasibility of delivering the intervention prototype and the barriers and enablers to be addressed to support intervention implementation and uptake by participants. This study was approved by the UCD Human Research Ethics Committee-Sciences (Ref no: LS-E-13-103-Hurley-Osing). Deductive thematic analysis based on Braun and Clarke’s method [38] was conducted on the data using two coding frames (feasibility and TDF, see Additional file 1). Table 1 shows the operational definitions of feasibility that were used in this study. Proposed changes to the intervention prototype were then addressed during a consensus building workshop outlined in step 4 below.

**Table 1:** Operational definitions of feasibility aspects related to intervention delivery [adapted from Bowen et al. [70]

Feasibility	Operational definition
Acceptability	The extent to which HSE physiotherapists consider the intervention prototype acceptable and appropriate within their service context
Demand	The extent to which HSE physiotherapists perceive the demand of delivering the intervention prototype, including identification of training needs
	The extent to which HSE physiotherapists perceive the demand of recruiting sufficient participants to the intervention within the feasibility trial
Practicality	The factors influencing the delivery of the intervention prototype in a range of HSE settings by a range of physiotherapists taking into account variations in staffing, equipment and facilities
Adaptation	The extent to which the intervention prototype content and delivery will need to be modified to enhance its acceptability and implementation in the feasibility trial
    - Physiotherapy managers (*n* = 10) completed a resource capacity checklist to identify the practicality of delivering the intervention prototype within their local service settings within the feasibility trial. An additional file shows this process in more detail (see Additional file 3).
    - The needs assessment provided the information needed to specify the SOLAS programme goals, the desired SM behaviours it would aim to change within participants and the discrepancies between the selected prototype and the additional content and theoretical underpinnings needed in SOLAS based on the literature and local needs. It also informed the feasibility and necessary modifications to primary care sites to support implementation of SOLAS in the planned trial.
- Step two: identification of outcomes, performance objectives and change objectives
  - The behavioural outcomes to be achieved by the SOLAS intervention were developed, and performance objectives (i.e. what a participant has to learn, do or change to achieve the specified outcomes) were stated for each behavioural outcome [32]. Using the information gathered from the needs assessment, the determinants of each behavioural outcome were identified and linked to relevant performance objectives creating a matrix of change objectives that detail what needs to change in the identified determinants to achieve the performance objective.
- Step three: selecting methods and practical applications
  - To operationalise the change objectives into practical applications, theoretically informed methods were selected, i.e. each determinant linked to a change objective was mapped to a TDF domain [39], and appropriate intervention methods (i.e. BCTs) were selected. BCTs are intervention components designed to influence the causal determinants that regulate behaviour [42]. This BCT identification process was conducted using appropriate literature [39, 40, 43], extensive discussion by the intervention development group and expert consultation (S Dean, L Atkins). The intervention prototype was reviewed for the specified BCTs, and any omissions were added to SOLAS. The selected BCTs were then converted into practical applications that could be implemented within SOLAS, taking into account the context and environment in which it was being delivered.
- Step four: creating an organised programme plan
  - A consensus building workshop was convened with physiotherapy stakeholders (*n* = 6 managers, 36 physiotherapists) working within all nine PCCC areas for final agreement on the adaptations needed to the intervention prototype structure to devise the SOLAS programme plan, as well as procedures to enhance implementation within the feasibility trial, i.e. physiotherapist training needs. Proposals on which consensus was reached (8/9 PCCC areas voted in favour) were incorporated into the SOLAS intervention design. The definitive intervention content and materials were adapted from the intervention prototype and relevant additions made.
- Step five: adoption and implementation plan
  - The programme use outcomes to achieve successful adoption by physiotherapy managers and implementation by clinical physiotherapists of the SOLAS intervention within the feasibility trial were specified. The determinants of programme adoption and implementation were identified from the TDF analysis of the qualitative studies within the needs assessment and linked to each performance objective to create a matrix of change objectives. The change objectives were converted into practical applications using a range of evidence-based BCTs [43, 44].
- Step six: creating an evaluation plan
  - The evaluation plan for SOLAS followed the recommended approach to establish the effect of the intervention on the target SM behaviours within a feasibility trial before moving to a definitive effectiveness trial [21]. This involved the specification of feasibility process and effect evaluation objectives, selection and development of indicators and outcome measures and a comprehensive feasibility trial design including treatment fidelity protocol. All procedures were tested in a pilot trial (UCD Human Research Ethics Committee-Sciences Ref no: LS-13-54-Currie-Hurley) to assess their acceptability and identify further adaptations during the development phase to enhance implementation during the feasibility trial. The pilot trial (April–Aug 2014) was run in four primary care health areas involving eight consenting physiotherapists and 20 consenting participants (12 F:8 M; mean (SD) age, 59.7 (8.9) years) and included individual semi-structured interviews with a sample of physiotherapists (*n* = 3) and participants (*n* = 5).

## Results

- Step one: needs assessment
  - The key findings of the multi-method needs assessment are provided below. An additional file shows these results in more detail (see Additional file 4).
  - Semi-structured interviews
    - The main themes from the manager interviews related to the TDF domains environmental context and resources (i.e. high caseload of patients with CLBP and OA requiring support to self-manage; important role but limited availability of psychologists to contribute to SM programmes), skills (staff experienced in running other groups), intention to support staff to set up group SM programmes and positive beliefs about the consequences of such programmes for patients and staff. The patients were positive about the experience of group physiotherapy (social influences), gained understanding of their condition (knowledge), skills and confidence in its SM (beliefs about capabilities), but would have liked it to be longer than 6 weeks (environmental context and resources) for further support.
  - Literature reviews
    - The consolidated definition of an intervention that promotes SM was designed to address both the process and outcomes related to SM that the SOLAS intervention could address (see Additional file 4). The rapid review found comparable effectiveness of physiotherapist-led group education and exercise interventions and individual physiotherapy or medical management for pain and disability outcomes in OA or CLBP [12]. Nonetheless, the high priority raised by physiotherapy managers to implement an evidence-based group SM programme rather than continuing with individual treatment and the putative beneficial effects of group-based SM programmes [19] confirmed our decision to develop a group SM programme that would meet the needs of the local population. From the rapid review, the FASA intervention [35] was selected as the prototype for adaptation that fulfilled our consolidated SM definition, being an education and exercise intervention based on the evidence-based ESCAPE programme for OA knee [45], designed for people aged over 50 years with OA hip, knee and/or lumbar spine, which has been found to be clinically effective compared to standard general practitioner (GP) care (personal communications, N Walsh). FASA was designed to be delivered by one physiotherapist in groups of up to eight people and considered acceptable and feasible to support SM by healthcare professionals in the UK [46]. In the FASA trial, it was delivered by trained research physiotherapists in UK healthcare settings and had not been previously delivered by health service physiotherapists in any jurisdiction including Ireland. We contacted the FASA intervention developer (N Walsh) who agreed to collaborate, provided and discussed the intervention materials, and allowed our team to observe its delivery in several UK settings. From this, we believed it had the potential to meet our target population and health service needs but would need formal evaluation to establish if it was fit for purpose, acceptable to Irish primary care physiotherapists and required adaptation prior to evaluation in the planned feasibility trial.
    - Within the most recent clinical guideline recommendations for OA and CLBP, the most consistent SM behaviours for programmes to promote/change within participants were a continuation or increase in physical activity, the use of joint specific exercise and pharmacological and non-pharmacological pain management approaches, with varying recommendations for healthy eating/weight management and pacing for OA and the use of active coping strategies for CLBP. The strategies that interventions should adopt to support SM behaviour ranged from none [5, 7] to highly specific [4, 47]. An additional file provides details of these findings (see Additional file 5). Three psychological factors that mediated (i.e. determinants) pain, disability and functional outcomes of interventions targeting these SM behaviours in chronic musculoskeletal pain were identified from the literature, i.e. increasing self-efficacy for OA and CLBP [48, 49], and reducing pain catastrophizing [48, 50] and fear [51] for CLBP. The literature reviews of behaviour change theories and techniques found variable integration in included studies, with social cognitive theory being the most frequently applied, and identified the most commonly used BCTs in group-based SM programmes as outlined in Additional file 4.
  - Focus groups
    - Following inter-rater reliability checks (>95 % agreement) [52], the focus groups resulted in 29 themes related to feasibility: programme participants (*n* = 5), content (*n* = 7), structure (*n* = 9) and delivery (*n* = 8). The most frequent theme was the feasibility of recruiting sufficient numbers of suitable participants, at the right time to participate, with varying views expressed on the optimal number for a successful group [6–14]. Opinions were mixed about the acceptability of including participants with CLBP, in addition to OA, and those below 50 years as within FASA [35], but considered essential to recruiting sufficient patients to ensure the intervention’s long-term viability. Physiotherapists were positive about the combined SM education and patient-led group exercise model of FASA, but felt 20 min was insufficient for education and discussion, 1 h was too short to run the group effectively, and two sessions per week as delivered in FASA while ideal was not acceptable from service or patient perspectives. An additional file provides further details of the feasibility analysis (see Additional file 6).
    - The findings of the barriers and enablers analysis identified 13 of the 14 TDF domains and 30 themes that predominantly related to the physiotherapists (*n* = 13) who would deliver the intervention, the target participants (*n* = 10), the intervention (*n* = 3), GPs (*n* = 2) and local organisations (*n* = 2). The majority of perceived barriers to delivering the intervention prototype were within the TDF environmental context and resources domain, beliefs about capabilities to deliver the intervention as intended and beliefs about its consequences. The key enablers were similar to the findings of the manager interviews. The significant influence of referring GPs as potential barriers and enablers to changing client attitudes, beliefs and expectations of the role of physiotherapy in promoting SM were also highlighted. From the participant perspective, the main barriers perceived by physiotherapists to be addressed were patients’ limited knowledge and skills in engaging in SM behaviours, particularly physical activity and exercise, low motivation to self-manage and regulate their behaviour and negative emotions about participating in a group. Further details of these findings are provided in an additional file (see Additional file 4).
    - The resource capacity checklist findings showed that most physiotherapy sites (95 %; *n* = 19) met the criteria to be considered eligible (≥60 %) to deliver the intervention prototype within existing capabilities or with essential modifications to facilities, equipment or staffing. Further details of these findings are provided in additional files (see Additional files 3 and 4).
    - Following this detailed needs assessment, the overall programme goal of SOLAS was defined as promoting SM behaviour for people with OA hip/knee, lumbar spine and/or CLBP in everyday life. The findings of the needs assessment informed several key decisions in designing the intervention. One, a number of determinants of the outcome of SM interventions in people with OA and CLBP identified from the literature (self-efficacy, motivation, catastrophizing, fear), focus groups (knowledge, skills, motivation, fear, behaviour-regulation) and expert consultation (behaviour regulation) were to be targeted within SOLAS (two of which were absent from FASA, i.e. catastrophizing, motivation; see Table 2) as outlined in Fig 2. Two, a specific behaviour change theory, self-determination theory (SDT), was selected to underpin participants’ uptake and engagement in the SOLAS intervention target behaviours as non-adherence to physical activity, exercise and diet is well recognised in the literature in these populations [53, 54]. SDT emphasises the importance of autonomy and autonomous self-regulation, core components of self-management behaviour [55–57]. According to SDT, social agents such as healthcare practitioners can influence an individual’s autonomous motivation for behaviour through their interpersonal style and interaction with the individual. A supportive interpersonal style satisfies an individual’s psychological need for autonomy, competence and relatedness leading to increased levels of autonomous motivation for the behaviour. Previously, SDT has been successfully applied to group-based education, exercise [58–61], physical activity [62], weight management [63], medication adherence [64], diabetes SM [65] and individual physiotherapy interventions [40, 66–68]. Several needs-supportive interpersonal strategies were identified from the literature to support physiotherapists’ effective delivery of the intervention using an SDT approach [58, 66, 67, 69] that would be operationalised during the physiotherapist training programme (step 5); e.g. providing meaningful rationale for SM behaviours, acknowledging participants’ feelings and perspectives and offering opportunities for participant input. Three, although the intervention prototype was found to be broadly consistent with current guidelines for OA, the SOLAS intervention would address the need for more evidence-based information on healthy weight, nutraceuticals and acupuncture [6]. Four, as FASA was not designed for non-specific CLBP, additional education content on the nature of CLBP, active coping strategies and current recommendations for acupuncture and TENS were needed. Finally, the education content required adaptation to reflect socio-demographic statistics related to physical activity, obesity, OA and LBP within the Irish population [3]. An additional file details the process of adapting the SOLAS intervention (see Additional file 5).

**Table 2:** Determinants of self-management behaviour and behaviour change techniques

Determinants of self-management behaviour	Needs assessment component that provided evidence of the determinant	TDF domain	Behaviour change techniques as per behaviour change technique taxonomy v1 (Michie et al. [42, 44])
Self efficacy	Systematic reviews of mediators for SM behaviour in CMP including OA, CLBP [48, 49]	Beliefs about capabilities To improve participants’ perceived competence to use each SM behaviour	1.2. Problem solving 2.2 Feedback on behaviour^a^ 2.3. Self-monitoring of behaviour 2.7 Feedback on outcome of behaviour 3.1. Social support (unspecified) 3.2. Social support (practical) 3.3. Social support (emotional) 8.1 Behavioural practice/rehearsal 8.7 Graded tasks 12.5 Adding objectives to the environment 15.1. Verbal persuasion about capability^a^
Catastrophizing^a^	Systematic reviews of mediators of CLBP outcomes [48, 50] CLBP guidelines [47]	Beliefs about consequences To reduce negative expectancies (catastrophizing) about consequences of engaging in specific SM behaviours	2.2 Feedback on behaviour^a^ 2.3. Self-monitoring of behaviour 2.7 Feedback on outcome of behaviour 5.1 Information re health consequences of behaviour 5.6 Information re emotional consequences of behaviour 9.1. Credible source
Fear	Systematic reviews of mediators of CLBP outcomes [51] CLBP guidelines [47] Focus groups barrier to patient participation to be addressed within intervention	Emotion To reduce negative emotional responses (fear) of specific SM behaviours	3.3 Social support—emotional 5.6 Information re emotional consequences of behaviour 11.2 Reduce negative emotions
Knowledge	Conceptual definition of SM [12] Focus groups barrier to patient participation to be addressed within intervention	Knowledge To increase participants knowledge for each SM behaviour	2.2 Feedback on behaviour^a^ 2.7 Feedback on outcome of behaviour 4.2 Information about antecedents of behaviour 5.1 Information about the health consequences of behaviour
Skills	Conceptual definition of SM [12] Focus groups barrier to patient participation to be addressed within intervention	Skills To develop participants proficiency to uptake each SM behaviour	1.1. Goal setting (behaviour) 1.2. Problem solving 1.3. Goal setting (outcome) 2.3. Self-monitoring of behaviour 4.1 Instruction on how to perform the behaviour 6.1. Demonstration of the behaviour 8.1 Behavioural practice/rehearsal 8.6 Generalisation of the targeted behaviour 8.7 Graded tasks 11.1 Pharmacological support 13.2 Framing/reframing 12.6 Body changes
Motivation^a^	Review of behaviour change theories [41], evidence of effectiveness in group interventions [58–61] and target patient and physiotherapist groups in Ireland [67, 68] Focus groups and manager interviews potential barrier to patient participation to be addressed within intervention	Intentions and goals To improve autonomous motivation of participants to engage in each SM behaviour	1.1 Goal setting (behaviour) 1.2 Problem solving 1.3. Goal setting (outcome) 1.4 Action planning 1.5 Review behavioural goal 1.6 Discrepancy between current behaviour and goal 1.7 Review outcome goal 1.8 Behavioural contract^a^ 3.1. Social support (unspecified) 3.2. Social support (practical) 3.3. Social support (emotional) 5.1 Information about health consequences 6.2 Social comparison 8.7 Graded tasks 9.1. Credible source 10.4 Social rewards
Behaviour regulation	Advice from behaviour change expert (S Dean) Focus groups barrier to patient participation to be addressed within intervention	Behavioural regulation Strategies to manage or change objectively observed or measured SM behaviour of participants	2.3 Self-monitoring of behaviour

^a^Absent from FASA
- Step two: identification of outcomes, performance objectives and change objectives
  - The specific intervention SM behavioural outcomes are:
    - i. To increase the physical activity level of participants
    - ii. To increase the use of evidence-based SM strategies by participants
  - Specific performance objectives were developed for the behavioural outcomes related to physical activity (*n* = 8) and use of SM strategies (*n* = 5) as detailed in Table 3. Using the information from step 1, the selected determinants were mapped to the performance objectives to articulate the specific change objectives of the intervention. For example, a performance objective for participants to ‘accept the benefits of physical activity’ was linked to the determinant of knowledge and resulted in a change objective ‘develops an understanding of the benefits of physical activity.’ Each change objective was written with an action verb followed by a statement of what is expected to occur as a result of the intervention [32]. An additional file shows this process in detail for all 13 performance objectives (see Additional file 7).

**Table 3:** Desired behavioural outcomes and performance objectives of the SOLAS intervention

Desired outcome 1: increases physical activity level of participants by the end of programme and 6-month follow-up	
Performance objective 1	Accepts the benefits of physical activity (PA)
Performance objective 2	Selects PA (s) relevant to lifestyle/pain condition
Performance objective 3	Performs selected PA(s)
Performance objective 4	Uses SMART goal setting for the selected PA(s)
Performance objective 5	Uses pacing to support selected PA (s)
Performance objective 6	Monitors progress in increasing PA
Performance objective 7	Copes with the challenges encountered with engaging in selected PA
Performance objective 8	Identifies long-term PA plan
Desired outcome 2: increase use of evidence-based self-management strategies by participants by end of programme and 6 month follow-up	
Performance objective 9	Accepts the role of SM approach
Performance objective 10	Selects appropriate evidence-based pain management strategies to self-manage pain condition
Performance objective 11	Uses pain coping strategies
Performance objective 12	Applies healthy eating guidelines for healthy lifestyle and to support weight management if appropriate
Performance objective 13	Uses specific exercise for pain condition
- Step three: selecting methods and practical applications
  - A full list of the selected BCTs and how they map to particular determinants is presented in Table 2. For example, the determinant self-efficacy along with the performance objective participants ‘perform selected physical activity’ was linked to the change objective, participants ‘improve self efficacy in ability to engage in selected physical activities’. The BCTs used to target this change objective ranged from ‘feedback’ and ‘self-monitoring of the behaviour’ to ‘behavioural practice’. These BCTs were translated into practical applications including group discussion and physiotherapist feedback on the previous week’s physical activity behaviour, a diary to self-monitor and review progress and opportunities to practice related activities in and outside the group. Table 4 provides a detailed description of how the selected BCTs were mapped to the change objectives and translated into a range of practical intervention applications.

**Table 4:** Intervention map linking change objectives to methods and practical applications

Change objectives and determinants of behaviour from TDF domains	Physical activity performance objectives	Class number	Content	Practical applications (mode of delivery)	Behaviour change techniques as per behaviour change technique taxonomy v1 (Michie et al. [42, 44])
Desired outcome 1: Increases physical activity level of participants by end of programme and 6 month follow-up					
KNOWLEDGE					
Develops an understanding of					
▪ the benefits of physical activity (PA) ▪ recommended types and levels of PA ▪ how to perform selected PA	PO.1, PO.3PO.2, PO.3	1–2	What are the benefits of exercise?; the Get Ireland Active physical activity recommendations; the current activity levels of Irish population according to age and chronic pain condition; what are appropriate exercises/walking/other physical activities	Lecture; group discussion; programme handbook; picture handout of exercises for home practice	2.2, 2.7, 4.2, 5.1, 9.1
▪ SMART goals and its relevance for PA	PO.4	1	What is SMART goal-setting?; how to complete a weekly goal setting sheet, review weekly goals and action plans	Lecture; group discussion; programme handbook	2.2, 2.7, 4.2, 5.1, 9.1
▪ pacing and its relevance for PA	PO.5	2	What is pacing and how to use it?	Lecture; group discussion; programme handbook	2.2, 2.7, 4.2, 5.1, 9.1
▪ tools for monitoring progress in PA	PO.6	1–2	How to use an activity plan; How to use a pedometer	Lecture; group discussion; programme handbook	2.2, 2.7, 4.2, 5.1, 9.1
▪ typical challenges while engaging in PA	PO.7	1,2,4,5	Factors influencing PA including fear of pain exacerbation	Lecture; group discussion; programme handbook	2.2, 2.7, 4.2, 5.1, 9.1
▪ available resources/facilities to support participation in PA	PO.8	6	What are the resources or facilities available to support long term PA participation	Local community resources handout	2.2, 2.7, 4.2, 5.1, 9.1
SKILLS					
▪ Develops physical skills to engage in PA	PO.3	1–6	Participation in supervised exercise class; participation in PA outside of the class	Instruction and demonstration by physiotherapist; practice by participant; programme handbook to record progress; feedback from physiotherapist	2.2, 2.7, 4.1, 6.1, 8.1, 8.6, 8.7, 10.4, 12.6
Develops skills to					
▪ apply relevant SMART goal setting to selected PA	PO.4	1–6	Weekly review of PA progress utilising goal setting and action planning	Group discussion; practice by participant during and outside of class; programme handbook to record progress; feedback from physiotherapist	1.1, 1.3, 2.2, 2.7, 2.3, 4.1, 6.1, 8.1, 10.4
▪ apply pacing to selected PA	PO.5	2	Physiotherapist provides instructions on how to pace	Instruction by physiotherapist; practice by participant outside of class; feedback from physiotherapist	1.2, 2.2, 2.7, 4.1, 6.1, 8.1, 8.6, 8.7, 10.4
▪ to monitor progress in increasing PA	PO.6	2–6	Weekly exercise diary in programme handbook; example of completed diary in programme handbook; pedometer provided to participants; demonstration of its use and written information in programme handbook	Instruction and demonstration by physiotherapist; practice by participant; programme handbook to record progress	1.1, 1.3, 2.3, 4.1, 6.1, 8.1
▪ to cope with the challenges encountered while engaging in selected PAs	PO.7	4–5	Practical relaxation session; instruction on how to select and use alternative methods to cope with pain (e.g. ice, heat, TENS)	Instruction by physiotherapist; practice by participant during and outside of class	4.1, 6.1, 8.1, 8.6
BELIEFS ABOUT CAPABILITIES					
Improves self-efficacy in ability to: ▪ perform selected PAs ▪ engage in selected PAs	PO.2PO.3	1–6	Review of previous week’s physical activity; participation in supervised exercise class	Group discussion; programme handbook to review progress; support from other participants; practice by participant; feedback from physiotherapist	1.2, 2.2, 2.3, 2.7, 3.1, 3.2, 3.3, 8.1, 8.7, 10.4, 12.5, 15.1
▪ use SMART goal setting	PO.4	1–6	Review of previous week’s goal-setting; instruction on how to set SMART goals and complete goal setting worksheet	Group discussion; programme handbook to review progress; support from other participants; practice by participant; feedback from physiotherapist	1.2, 2.2, 2.3, 2.7, 3.1, 3.2, 3.3, 6.1, 8.1, 8.7, 12.5, 15.1
▪ use pacing	PO.5	2–6	Review participants’ attempts to pace; Instruction on how to pace	Group discussion; support from other participants; practice by participant; feedback from physiotherapist	1.2, 2.2, 2.3, 2.7, 3.1, 3.2, 3.3, 6.1, 8.1, 8.7, 12.5, 15.1
▪ use tools to monitor progress	PO.6	2–6	Review participants’ attempts to use tools including pedometer; instruction on how to use monitoring tools	Practical demonstration of pedometer; practice by participant; programme handbook to review progress; feedback from physiotherapist	1.2, 2.2, 2.3, 2.7, 3.1, 3.2, 3.3, 6.1, 8.1, 8.7, 12.5, 15.1
▪ cope with challenges encountered during PA participation	PO.7	2–6	Factors influencing PA including fear of pain exacerbation; review participants’ attempts to select and use alternative methods to cope with pain (e.g. ice, heat, TENS); instruction on how to use alternative methods	Group discussion; practice by participant; feedback from physiotherapist	1.2, 2.2, 2.3, 2.7, 3.1, 3.2, 3.3, 6.1, 8.1, 8.7, 15.1
▪ engage in long-term PA	PO.8	6	Review participants progress over the course of the programme. Provide information regarding community resources to support activity maintenance	Group discussion; support from other participants; feedback from physiotherapist; community resource leaflet	1.2, 2.2, 2.3, 3.1, 3.2, 3.3, 6.1, 8.1, 8.7, 15.1
BELIEFS ABOUT CONSEQUENCES					
Reduce pain catastrophizing beliefs’ related to consequences of engaging in PA	PO.2, PO.3, PO.7, PO.8	1–6	Information on pain related to physical activity participation [wks 2,5]; review of previous week’s physical activity; participation in supervised exercise class	Lecture; group discussion; programme handbook to review progress; practice by participant; feedback from physiotherapist	2.2, 2.3, 2.7, 5.1, 5.6, 9.1, 10.4
EMOTION					
Reduce fear related to engaging in selected PAs	PO.3, PO.7, PO.8	1–6	Information on pain related to physical activity; review of previous week’s physical activity; participation in supervised exercise class	Lecture; group discussion; social support from other participants, practice by participant; feedback from physiotherapist	2.2, 2.7, 2.3, 3.3, 5.1, 5.6, 8.1, 9.1, 11.2,
INTENTIONS AND GOALS					
▪ Increase autonomous motivation to engage in selected PA ▪ Increase autonomous motivation to engage in long-term PA	PO.1, PO.2, PO.3PO.8	1–6	What are the benefits of exercise?; the Get Ireland Active physical activity recommendations; the current activity levels of Irish population according to age; what are appropriate exercises/walking; review of weekly progress	Lecture; group discussion; social support from other participants; programme handbook to record physical activity goal; feedback from physiotherapist	1.1, 1.2, 1.3, 1.4, 1.5, 1.6, 1.7, 1.8, 3.1, 3.2, 3.3, 5.1, 6.2, 9.1, 10.4
Increase autonomous motivation					
▪ to use SMART goal setting	PO.4	1–6	What is SMART goal-setting?; how to review weekly goals and action plans; review of weekly progress	Lecture; group discussion; programme handbook; social support from other participants; feedback from physiotherapist	1.1, 1.2, 1.3, 1.4, 1.5, 1.7, 3.1, 3.2, 3.3, 5.1, 6.2, 9.1, 10.4
▪ to use pacing	PO.5	2	What is pacing and how to use it	Lecture; group discussion; feedback from physiotherapist	1.1, 1,2, 1.3, 1.4, 1.7, 3.1, 3.2, 3.3, 5.1, 6.2, 8.7, 9.1, 10.4,
▪ to use tools to monitor progress in PA	PO.6	2–6	How to use an activity plan; how to use a pedometer; review participants’ attempts to use tools	Lecture; group discussion; programme handbook; feedback from physiotherapist	1.1, 1.2, 1.3, 1.4, 1.7, 1.8, 3.1, 3.2, 3.3, 5.1, 6.2, 9.1, 10.4
▪ to cope with challenges encountered during PA participation	PO.7	1–6	Factors influencing PA including fear of pain exacerbation; review participants’ attempts to select and use alternative methods to cope with pain (e.g. ice, heat, TENS); instruction on how to use alternative methods	Lecture; group discussion; feedback from physiotherapist	1.1, 1.2, 1.3, 1.4, 1.7, 1.8, 3.1, 3.2, 5.1, 6.2, 8.7, 9.1, 10.4
BEHAVIOURAL REGULATION					
▪ Develops ability to incorporate and monitor effects of PA into daily life	PO.3, PO.5	1–6	Weekly activity diary record	Programme handbook to record progress	2.3
▪ Develops ability to implement tools to monitor PA progress	PO.6	2–6	Weekly activity diary record	Programme handbook to record progress	2.3
Desired outcome 2: increase use of evidence-based self-management strategies by participants by end of programme and 6-month follow-up					
Change objectives and determinants of behaviour from TDF domains	Self-management performance objectives (PO)	Class number	Content	Practical applications [mode of delivery]	Behaviour change techniques as per behaviour change technique taxonomy v1 (Michie et al. [42, 44])
KNOWLEDGE					
Develops an understanding of					
▪ the rationale for self-management	PO.9	1	Aims and structure of the programme; prevalence, pathology and prognosis of OA and CLBP and their relevance to self-management. Application of self-management strategies	Written patient information leaflet; programme handbook; lecture; group discussion Group discussion	2.2, 2.7, 4.2, 5.1, 9.1
▪ evidence-based pharmacological and non-pharmacological pain management strategies relevant to their pain condition	PO.10	2, 4	Factors influencing pain; pain gate theory; safe application of ice, heat. Evidence for TENS, acupuncture. Drug pyramid of analgesic, anti-inflammatory, opioid and steroid medication; rationale for selection of approaches	Lecture; programme handbook Group discussion	2.2, 2.7, 4.2, 5.1, 9.1, 11.1
▪ pain coping strategies	PO.11	2, 5	Factors influencing pain; anxiety, mood and pain; relaxation techniques; progressive muscular relaxation	Lecture, group discussion, programme handbook, practical relaxation session	2.2, 2.7, 4.2, 5.1, 5.6, 9.1
▪ healthy eating guidelines and healthy weight	PO.12	3	Prevalence of obesity in Ireland; relationship to joint and back pain, life expectancy; balanced weight, waist size for low, moderate, high risk, relationship to physical activity and its measurement; healthy eating guidelines; food pyramid, eatwell plate, portion size, food and exercise	Lecture; group discussion; programme handbook; practical demonstration of waist measurement	2.2, 2.7, 4.2, 5.1, 9.1
▪ how to perform selected specific exercises	PO.13	1–6	Types of specific exercises and their effects	Lecture, programme handbook, practical demonstration of exercises	2.2, 2.7, 4.2, 5.1, 9.1
SKILLS					
Develop skills to appropriately:					
▪ select and use evidence-based pharmacological and non-pharmacological pain management strategies relevant to their pain condition	PO.10	4–6	Reflection on ability and outcome of previous weeks selection and use of pharmacological and non-pharmacological pain management approaches relevant to their pain condition	Group discussion; feedback from the physiotherapist	1.2, 2.2, 2.7, 4.1, 6.1, 10.4, 11.1
▪ select and use pain coping strategies	PO.11	5–6	Reflection on ability and outcome of selection and use of pain coping strategies	Group discussion, programme handbook Feedback from the physiotherapist Practical class in relaxation skills	1.2, 2.2, 2.7, 4.1, 6.1, 8.1, 10.4, 13.2
▪ follow healthy eating guidelines and monitor weight	PO.12	3–4	Reflection on ability and outcome of monitoring healthy eating and weight	Group discussion; programme handbook	1.2, 2.3, 4.1, 6.1, 8.1
▪ engage in specific exercises	PO.13	1–6	Supervised group exercise class	Participation in exercise class and peer observation; discussion with physiotherapist during exercise session, group discussion	1.1, 1.2, 2.2, 2.3, 2.7, 4.1, 6.1, 8.1, 8.7, 12.6
BELIEFS ABOUT CAPABILITIES					
Increase self-efficacy in ability to:					
▪ use evidence-based pharmacological and non-pharmacological pain management strategies relevant to their pain condition	PO.10	4–6	Reflection on ability and outcome of previous weeks selection and use of pharmacological and non-pharmacological pain management approaches relevant to their pain condition	Group discussion and feedback from physiotherapist	1.2, 2.2, 2.7, 3.1, 3.2, 3.3, 10.4, 15.1
▪ apply pain coping strategies	PO.11	5–6	Reflection on ability and outcome of selection and use of pain coping strategies	Group discussion and feedback from physiotherapist	1.2, 2.2, 2.7, 3.1, 3.2, 3.3, 8.1, 10.4, 15.1
▪ follow healthy eating guidelines and monitor healthy weight	PO.12	3–4	Reflection on ability and outcome of monitoring healthy eating and weight and use of tools to support this	Group discussion and feedback from physiotherapist	1.2, 2.2, 2.3, 3.1, 3.2, 3.3, 10.4, 12.5, 15.1
▪ engage in specific exercises	PO.13	1–6	Supervised group exercise class Practice of selected exercises at home; provision of theraband to support exercises at home	Participation in self-selected exercises and progressions with support and feedback from physiotherapist. Peer observation and discussion. Completion of weekly exercise diary in class	1.2, 2.2, 2.3, 2.7, 3.1, 3.2, 3.3, 8.1, 8.7, 10.4, 12.5, 15.1
BELIEFS ABOUT CONSEQUENCES					
▪ Reduce pain catastrophizing beliefs’ associated with pain condition by using pain coping strategies	PO.10, PO.11	2–6	Review of previous week	Group discussion; discussion with physiotherapist	2.2, 2.3, 2.7, 5.1, 5.6, 9.1, 10.4
▪ Reduce pain catastrophizing beliefs’ related to consequences of engaging in specific exercises	PO.13	1–6	Supervised group exercise class	Participation in exercises, peer observation and group discussion Review of outcome of previous weeks home exercises programme; discussion with physiotherapist	2.2, 2.3, 2.7, 5.1, 5.6, 6.1, 8.1, 9.1
EMOTION					
▪ Reduce fear associated with pain condition by using pain coping strategies	PO.10, PO.11	2–6	Pain and factors influencing it; review of previous week	Lecture; group discussion and feedback	2.2, 2.3, 2.7, 3.3, 5.6, 9.1, 11.2
▪ Reduces fear related to engaging in selected specific exercises	PO.13	1–6	Supervised group exercise class	Participation in exercises; peer observation and group discussion	2.2, 2.3, 2.7, 3.3, 5.6, 8.1, 9.1, 11.2
INTENTIONS AND GOALS					
Increase autonomous motivation to:					
▪ self-manage their pain condition ▪ use evidence-based pharmacological and non-pharmacological pain management strategies relevant to their pain condition	PO.9, PO.10	2–6	Factors influencing and easing pain; review of previous week; planning for subsequent week to select and use relevant approaches	Lecture; group discussion led by physiotherapist	1.2, 3.1, 3.2, 3.3, 5.1, 6.2, 9.1, 10.4
▪ to use pain coping strategies	PO.9, PO.11	2–6	Factors influencing and easing pain; review of previous week; planning for subsequent week to select and use relevant approaches	Lecture; group discussion led by physiotherapist	1.2, 3.1, 3.2, 3.3, 5.1, 6.2, 8.7, 9.1, 10.4
▪ to follow healthy eating guidelines and to monitor healthy eating and weight	PO.9, PO.12	3	Effect of diet and weight on pain Review of previous week, planning for subsequent week to select and use relevant strategies	Lecture; programme handbook; group discussion led by physiotherapist	1.1, 1.2, 1.3, 1.4, 1.5, 1.7, 3.1, 3.2, 3.3, 5.1, 6.2, 9.1, 10.4
▪ to perform selected specific exercises	PO.9, PO.13	1–6	Effect of specific exercise on joint and back pain; supervised group exercise class; home exercise programme	Lecture; programme handbook; group discussion; participation in group exercise class and home exercise programme; peer observation and discussion	1.1, 1.2, 1.3, 1.4, 3.1, 3.2, 3.3, 5.1, 6.2, 8.7, 9.1, 10.4
BEHAVIOURAL REGULATION					
▪ Develop ability to monitor pain condition to select and apply evidence-based pharmacological and non-pharmacological pain management strategies relevant to their pain condition	PO.10	2–6	Weekly activity diary; record of pain-related symptoms	Programme handbook	2.3
▪ Develop ability to monitor pain condition to select and apply appropriate pain coping strategies	PO.11	2–6	Weekly activity diary; record of pain-related symptoms	Programme handbook; use of relaxation CD at home	2.3
▪ Develop ability to monitor healthy eating and weight	PO.12	3–6	Food and exercise diary Measurement of waist size demonstrated by physiotherapist	Programme handbook Tape measure provided	2.3
▪ Develop ability to incorporate and monitor effects of specific exercise in daily life	PO.13	1–6	Weekly specific exercise diary	Programme handbook Completion of weekly exercise diary in class	2.3
- Step four: creating an organised programme plan
  - The consensus building workshop held nine ballots for proposed adaptations to the FASA prototype structure, physiotherapist training and participant recruitment procedures of which eight were carried (Table 5). It was agreed that the definitive SOLAS intervention would comprise six weekly sessions of 90 min (45 min education/discussion and 45 min exercise) for people aged at least 45 years to be delivered by one physiotherapist in groups of four to eight participants with OA of the hip, knee, lumbar spine and CLBP. The adapted education content was incorporated into the new structure (Table 6), and new programme materials were adapted from FASA (i.e. intervention slides and script, participant programme handbook, exercise photographs of an age appropriate model). A review of FASA for evidence-based materials to enhance physical activity, healthy eating, weight management and pain coping strategies (see Additional file 5) identified the need for additions to SOLAS as indicated in Table 6.

**Table 5:** Consensus building workshop results

Points for consensus	Votes in favour^a^	Resulting actions
Do you agree that a 6-week programme with weekly sessions of 1.5 h is feasible in your service area?	8/9 88.9 %	Programme structure (6 weekly sessions, 1.5 h per session) was adopted
Do you agree that the proposed group class structure and size (stop/start programme with 6–8 participants at week 1) is feasible for the purposes of the trial in your service area? Site A Concern over the lone worker and staff: client ratio Query students required for assistance with high risk patient group Site B We would prefer 8 = 12 with 2 physios. Will be running class in community centre. Want 2 physios for flexibility to keep running in case of absence	7/9 77.8 %	Concerns of one site re lone worker addressed by provision of second support worker which raised agreement threshold to 88.9 %
Do you agree that a minimum age of 45 years for inclusion in the trial programme is feasible in your service area?	8/9 88.9 %	Minimum age limit of 45 years was adopted.
Do you agree that the following administrative procedures related to participant recruitment are feasible for your physiotherapy team:		
Raise awareness of the feasibility trial amongst primary care teams	8/9 88.9 %	HSE community managers/physiotherapists will raise awareness of the trial at primary care team meetings or in correspondence.
Identify suitable referrals (i.e. screen referral letters and wait list) for the feasibility trial	9/9 100 %	HSE community physiotherapists will screen the referral letters and waiting list to support recruitment by identifying potentially eligible clients.
Send standard invitation letter with added description of the feasibility trial and invitation for client to contact the study team	9/9 100 %	The PCCC physiotherapist will send invitation letters to clients, which describes the study and invites interested clients to contact the UCD research team. Admin support will be provided by the UCD research team to support this aspect of recruitment.
See patients for 15 min post eligibility screening for patient education concerning the pathophysiology of their condition.	3/9 33.3 %	PCCC physiotherapists agree that the assessment by the UCD research physiotherapist is sufficient to allow access to the group. The 15-min post eligibility screening for patient education was not considered feasible by the PCCC physiotherapists. Education on pathophysiology will be incorporated into the intervention.
Do you agree that it is feasible to have one physiotherapist deliver each group within the intervention in your service area for the purposes of the trial and to agree the role of any additional support staff with the research team in advance of your participation in the trial?	9/9 100 %	It was agreed that one physiotherapist would deliver all components of the group. A second person could play a supportive role where required for reasons of safety. This second person could be a physiotherapist, student, fitness instructor or other healthcare professional. The role of this second person must be agreed with the research team in advance of participation in the trial.
Do you agree that is it feasible for physiotherapists in your service area to allocate time to participate in 1.5 days of training (as outlined), plus a 2-h site visit in advance of your participation in the trial?	9/9 100 %	The training plan was agreed and adopted.

^a^An a priori definition of consensus was established as 80 % of the vote in favour of any issue (e.g. 8/9 local health areas had to vote in favour)

**Table 6:** Comparison of FASA and SOLAS interventions

FASA intervention [35]		SOLAS intervention [36]^a^	
Class structure			
▪ 12 classes, twice weekly × 60 min × 6 weeks ▪ 15–20 min: review of participants progress since last session, weekly education topic—introduction by physiotherapist and group discussion ▪ 40–45 min: supervised group exercise		▪ 6 classes, once weekly × 90 min × 6 weeks ▪ 10 min: recap of previous session and review of participants’ progress towards achieving their weekly goal ▪ 25 min: weekly education topic—introduction by physiotherapist and group discussion ▪ 45 min: supervised group exercise ▪ 10 min: after exercises session review and participant action planning for weekly goal	
Education and materials			
Class		Class	
1	Education: aims of the programme, cycle of change, review of FASA exercise programme Materials: participant programme handbook	1	Education: aims of programme, *back pain* and OA causes, cycle of change, exercise recommendations, *physical activity levels in Ireland*, benefits of exercise/physical activity, review of SOLAS exercise programme, and goal setting Materials: participant programme handbook
2	Joint pain and benefits of exercise, exercise recommendations		
3	Goal setting and action plans	2	Education: activity-rest cycle and pacing activities, *use of pedometer, walking technique*, understanding pain, physical activity diary, goal setting and action plan Materials: *Yamax SW-200 Pedometer*
4	Activity-rest cycle and pacing activities		
5	Healthy diet	3	Education: *balanced weight, obesity levels in Ireland*, healthy eating, *portion size, measuring waist circumference*, physical activity diary, goal setting and action plan, food and drink diary Materials: tape measure, *Your Guide to Healthy Eating using the Food Pyramid, 101+ Square Meals*
6	Heat and ice		
7	Mid-way review	4	Education: mid-way review, evidence-based pain management with ice/heat, medication, TENS, acupuncture, physical activity diary, goal setting and action plan
8	Anxiety, mood and pain		
9	Relaxation techniques	5	Education: anxiety, mood and pain, managing flare-ups, *pain coping strategies*, relaxation techniques and practice, physical activity diary, goal setting and action plan Materials: *relaxation CD*
10	Drug management, dietary supplements, TENS, acupuncture		
11	Managing flare-ups	6	Education: discharge planning, maintaining a good exercise routine in the long-term, *local resources to support physical activity information*, long-term physical activity diary, final goal setting and action plan, programme feedback Materials: *local resources to support physical activity leaflet, graduation certificate*
12	Exercising in the long-term		

Supervised group exercise: range of general aerobic (*n* = 8: step ups, stationary cycling) and joint specific mobility and strengthening exercises for the lumbar spine (*n* = 4), hip (*n* = 6) and knee (*n* = 4) designed to increase participants’ participation in exercise and physical activity. The frequency and number of exercise stations completed is determined by each participant with support from the physiotherapist if needed

^a^Additions to SOLAS intervention in italics
- Step five: adoption and implementation plan
  - The programme use outcomes are:
    - i. PCCC physiotherapy managers adopt the SOLAS intervention and participant recruitment procedures.
    - ii. PCCC physiotherapists implement the SOLAS intervention and participant recruitment procedures.
  - The specific performance objectives for each programme use outcome are presented in Table 7. The determinants of physiotherapist behaviour identified from the needs assessment were mapped to the performance objectives to articulate the specific change objectives. An additional file shows the matrix of change objectives in detail (see Additional file 8). A range of theoretically derived BCTs and practical strategies were selected by the intervention development group to target the change objectives of adoption and implementation as detailed in Table 8. For example, in order to influence the determinants physiotherapists’ knowledge, skills, beliefs about capabilities and beliefs about consequences to deliver the SOLAS intervention linked to the performance objective physiotherapists ‘complete training in the delivery of the SOLAS intervention’, a bespoke training programme underpinned by selected BCTs was developed.

**Table 7:** Programme use outcomes and performance objectives for adoption and implementation

Adoption use outcome: physiotherapy managers adopt the SOLAS intervention and participant recruitment procedures within their PCCC service area	
Performance objective 1	Agree to allow their physiotherapy service to participate in the SOLAS feasibility trial
Implementation use outcome: physiotherapists implement the SOLAS intervention and participant recruitment procedures within their PCCC service area	
Performance objective 2	Agree to participate in the SOLAS feasibility trial
Performance objective 3	Complete training in the content and delivery of the SOLAS intervention
Performance objective 4	Prepare local site to support delivery of the SOLAS intervention
Performance objective 5	Support participant recruitment to the SOLAS intervention within the feasibility trial

**Table 8:** Programme adoption and implementation of SOLAS intervention and participant recruitment linking change objectives to practical applications

Change objectives and TDF domain	Performance objective	Behaviour change techniques chosen to address each TDF domain based on Michie et al. [43, 44]. The listed codes and terms for each technique are based on BCTV1 taxonomy [42]	Practical applications
ENVIRONMENTAL CONTEXT AND RESOURCES			
Managers allocate resources to support service to deliver SOLAS intervention	PO.1	12.1 Restructuring the physical environment (in sites scoring <80 % on the resource checklist)	Managers approve the redeployment/purchase of equipment to allow delivery of the SOLAS intervention Managers approve rental of leisure centre facilities to deliver the SOLAS intervention if clinic space unsuitable
INTENTIONS Managers provide written agreement to allow their service to participate in SOLAS feasibility trial	PO.1	1.8 Behavioural contract	Written agreement from each physiotherapy manager to: i. allow their service area to participate in the feasibility trial and deliver treatment according to randomisation ii. support participant recruitment to SOLAS feasibility trial iii. nominate two physiotherapists to attend training if randomised to the SOLAS intervention arm
Physiotherapists agree to participate in the SOLAS feasibility trial	PO.2	1.8 Behavioural contract	Written agreement obtained from each physiotherapist to participate in the SOLAS feasibility trial which involves: i. Screening the waiting list for potentially eligible participants ii. Sending a standardised invitation letter to clients with support from the UCD research team iii. Supporting the UCD research team in efforts to reach recruitment targets iv. Attending and participating in training provided by research team v. Providing treatment according to the random assignment of your site vi. Documenting treatment provided vii. Allowing audio recording and direct observation of treatment to assess fidelity viii. Participating in an individual semi-structured interview if randomised to provide group treatment at the end of delivery.
KNOWLEDGE Develops an understanding of the structure, content and materials within the SOLAS intervention	PO.3	5.1 Information about health consequences of the intervention	Physiotherapist training programme^a^ Pre-reading information about intervention content and structure, selected research papers, brief power point lectures, physiotherapist training handbook, power point slides with script of intervention content, discussion about beliefs about consequences of the intervention components. On completion of training, physiotherapists receive copy of all intervention materials listed in Table 6.
Develops an understanding of the needs supportive interpersonal delivery style of the SOLAS intervention		5.3 Information about social and environmental consequences of the intervention	Pre-reading information about underpinning behaviour change theory, selected research papers, brief power point lectures, physiotherapist training handbook, discussion about beliefs about consequences of delivering the intervention the using a needs supportive interpersonal style strategies to *support participant autonomy for the behaviour*: • Offer a meaningful rationale for the particular behaviour • Provide opportunities for input and choice to participants • Use support and encouragement rather than pressurising behaviour *support participant competence to engage in the behaviour*: • Set clear expectations and provide appropriate direction • Provide positive and information rich feedback • Provide participants with opportunities to practice behaviours • Use collaborative goal-setting, action planning and problem solving *support relatedness by encouraging interpersonal involvement* • Build relationships with participants (e.g. proximity, using names, etc.) and between participants • Acknowledge and take into account participants’ feelings and perspectives
SKILLS			
Develops skills in delivering the SOLAS intervention	PO.3	1.1 Goal setting (behaviour) 1.4 Action planning	Goal setting exercise—physiotherapists individually reflect and set a goal and action plan related to practising delivery of the needs supportive strategies discussed in training in their everyday clinical practice
		1.2 Problem solving	Workshop and reflection during/after day 1 training on the challenges and possible solutions to delivering the intervention at their site for discussion with course facilitators and peers on day 2
		2.2 Feedback on behaviour	Verbal feedback by research team during training; role play of delivering the intervention using a needs supportive interpersonal style. Verbal and written feedback of training audio-recording of delivering the intervention using needs supportive interpersonal style strategies by research team following training
		2.3 Self-monitoring of behaviour	Audio-recording of delivery of components of the intervention during training and self-rating of quality of delivery using a needs supportive interpersonal style following training
		4.1 Instruction on how to perform the behaviour (i.e. deliver the intervention)	Brief power point lecture, physiotherapist training handbook
		6.1 Demonstration of the behaviour 6.2 Social comparison	Video examples of delivering components of the intervention, e.g. good and poor practice goal setting, problem solving, giving feedback. Encouragement of physiotherapists to compare their use of a needs supportive interpersonal style with the video examples and their peers within the training programme
		8.1 Behavioural practice of delivering elements of the intervention	Role play, peer observation and feedback, group discussion of delivering the intervention using a needs supportive interpersonal style
		8.7 Graded tasks	Graded role play activities delivering the intervention using a needs supportive interpersonal style, i.e. simple one to one interactions progressing to microteaching activities delivering a component of the class to a group of peers
BELIEFS ABOUT CAPABILITIES			
Improve confidence in ability to deliver the SOLAS intervention	PO.3	1.2 Problem solving	Workshop: physiotherapists estimate the number of exercise stations that could be provided in their clinic space and equipment, and identify the need for changes to the clinic space/equipment to support delivery of the intervention
		2.2 Feedback on behaviour during training	Verbal feedback by research team during training; role play of delivering the intervention using a needs supportive interpersonal style. Verbal and written feedback of training audio-recording of delivering the intervention using a needs supportive interpersonal style by research team following training
		2.3 Self-monitoring of behaviour during training	Audio-recording of delivery of components of the intervention during training and self-rating of quality of delivery using a needs supportive interpersonal style following training
		3.1 Social support (unspecified)	Encouragement from facilitator and peers for delivery of the intervention as intended during role play in training
		3.2 Social support (practical)	Practical support from facilitator in supporting delivery of the intervention following training by provision of written feedback from audiorecordings
		6.1 Demonstration of the behaviour	Video examples of delivering components of the intervention, e.g. good and poor practice goal setting, problem solving, giving feedback
		8.1 Behavioural practice/rehearsal	Role play, peer observation and feedback, group discussion of delivering the intervention using a needs supportive interpersonal style
		8.7 Graded tasks	Graded role play activities delivering the intervention using a needs supportive interpersonal style, i.e. simple one to one interactions progressing to microteaching activities delivering a component of the class to a group of peers
		15.1 Verbal persuasion to boost self-efficacy to deliver the intervention using a needs supportive interpersonal style	Course facilitator with expertise in self-determination theory verbally persuades physiotherapists they can successfully deliver the SOLAS intervention using a needs supportive interpersonal style following training, and argues against self-doubts
		15.3 Focus on past success	Group discussion on past experience in managing clients with chronic musculoskeletal pain, and/or delivering groups
BELIEFS ABOUT CONSEQUENCES			
Reduce concerns about potential for clients to experience flare-ups during the SOLAS intervention	PO.3	5.1 Information on the health consequences of engaging in physical activity	Brief power point lecture about managing flare-ups, physiotherapist training handbook, relaxation CD, discussion about beliefs about consequences clients experiencing flare-ups
		9.1 Credible source	Course facilitator with expertise in chronic musculoskeletal pain management presents verbal and visual information from the literature concerning flare-ups and their management in the context of the SOLAS intervention
ENVIRONMENTAL CONTEXT AND RESOURCES			
Improve physical environment to prepare for delivery of the SOLAS intervention	PO.4	12.1 Restructuring the physical environment (in sites scoring <80 % on the resource checklist) 12.5 Adding objects to the environment	Site visit by research team to provide advice on selection of appropriate room within clinic space or local leisure centre to deliver the education and exercise components of the intervention (including set-up of chairs/projector/laptop) Research team make recommendations for redeployment/purchase of equipment within available resources (laptops, exercise machines) to allow delivery of the intervention Research team provide colour laminates of each individual exercise of an age appropriate model to display during the intervention Research team provide USB of intervention slides, handout with suggested script for each slide, programme handbook, programme materials.
SOCIAL INFLUENCES			
Adapt social environment to prepare for delivery of the SOLAS intervention	PO.4	12.2 Restructuring the social environment	To ensure consistency across sites in delivery of the SOLAS intervention, one trained physiotherapist to deliver the intervention, but a second staff member (i.e. physiotherapist, student) can support delivery if concerns about safety.
Increases awareness of the SOLAS intervention, feasibility trial and participants recruitment pathway to referring GPs, primary care team and potential participants	PO.5	12.2 Restructuring the social environment (referral and screening procedures for potential participants to the trial)	Physiotherapists/managers will raise awareness of the SOLAS intervention within the feasibility trial with referring GPs at primary care meetings and relevant correspondence Screening of GP referrals to identify potentially suitable participants for the SOLAS intervention by physiotherapists Physiotherapists will send invitation letter to potential participants referred by GPs to raise awareness of the feasibility trial Trial website to increase awareness of the trial to potential participants and referring GPs
KNOWLEDGE			
Develop GPs understanding of the participant recruitment pathway to the SOLAS intervention and feasibility trial	PO.5	5.1 Provide GPs with information about health consequences of the SOLAS intervention and of clients who have agreed to participate in the feasibility trial	Letter to GPs giving information about the feasibility trial, content of SOLAS intervention and control arms and eligibility criteria Letter to GPs when clients they refer become participants in the trial

^a^Two day training programme [i.e. 12 hrs] small group training course [up to 8 PTs], designed and co-facilitated by the intervention developers; a Physiotherapist and senior researcher (DAH) who holds an MSc in Musculoskeletal Physiotherapy and a PhD in back pain research, and a registered Psychologist and researcher (JM) who holds an MA in Organisational and Social Psychology and a PhD in Sport and Exercise Psychology
- Step six: creating an evaluation plan
  - A cluster randomised controlled feasibility trial has been designed to evaluate SOLAS (Current Controlled Trials ISRCTN49875385, 26^th^ March 2014) [36]. A cluster randomised trial design was chosen to avoid contamination of the control group [25]. The most appropriate comparison was considered usual treatment [20], defined as individual physiotherapy care. The trial aims to assess the acceptability and demand of the SOLAS intervention to patients and physiotherapists compared to usual treatment [70], the feasibility of trial procedures and the most efficient and effective study design for a definitive trial. In the absence of a suitable validated SM outcome measure from the literature [12, 71, 72], a new measure was developed for evaluation within the feasibility trial. A range of effect and mediation outcome measures were selected from the literature to be evaluated within the trial. A detailed fidelity protocol has been developed and published separately [73]. The pilot trial resulted in further minor adaptations to the intervention content and materials, enhanced physiotherapist training from 1.5 to 2 days (more emphasis on goal setting, problem solving, and feedback) and amended participant eligibility criteria (CLBP participants age ≤30 years) prior to commencement of the main feasibility trial in September 2014.

## Discussion

This study provides a detailed example of the systematic application of the IM protocol to develop the SOLAS theory-driven evidence-based group intervention to promote self-management in people with OA hip/knee and/or CLBP through adaptation of an existing evidence-based programme. There is currently limited literature on the detailed reporting of the critical development phase of complex interventions in primary healthcare and the application of IM in chronic musculoskeletal pain or physiotherapy, and this study should inform future researchers in this evolving field. We followed all the recommended steps within IM [32], engaged a representative sample of stakeholders using a mix of qualitative and quantitative methods, applied emerging behaviour change methodologies to inform SOLAS intervention development and implementation and adhered to TIDieR guidance in its description [26, 74]. We believe that the decision to adapt an existing intervention enhanced its uptake by stakeholders, the quality of the intervention and materials and allowed the intervention development group to address the practicalities of implementation, including physiotherapist training from the outset.

The SOLAS intervention provides for the first time a group intervention for people with two of the most common chronic musculoskeletal conditions (i.e. OA and CLBP) presenting to primary care. While the multi-joint aspect of the FASA prototype for people with OA aged over 50 years was acceptable to UK physiotherapists [46], and credible to Ireland’s stakeholder primary care physiotherapists, it was considered necessary to adapt the diagnostic pool for SOLAS to include people with non-specific CLBP aged at least 30 years to increase its acceptability to meet their service needs. Further adaptations were required to implement recent clinical guideline recommendations for OA and CLBP and Irish sociodemographic statistics. Finally, the overall structure of the programme was adapted from 12 twice weekly, 1-h sessions to 6 once weekly, 90-min sessions despite some patients and physiotherapists expressing support for a longer programme. Nonetheless, the majority of physiotherapists believed that 6 weeks reflected current practice and was more realistic for patients, which is supported by a recent systematic review [75]. However, it has been proposed that longer programmes may provide larger treatment effects [13, 14, 75], which could be considered worthwhile by patients [76]. Similarly, the decision to deliver the intervention once rather than the more frequent twice weekly reported in the literature [75] was taken to enhance acceptability to local physiotherapist stakeholders as demonstrated in a quote from one focus group participant ‘twice a week is…a nice idea. What you use in trials and then never use in practice’. The feasibility trial results will inform whether these decisions were correct and reflect the reality of collaborating with healthcare professional stakeholders in developing interventions while also taking account of the evidence. If positive, this pragmatic example of involving clinicians has the potential to enhance future knowledge translation of evidence-based interventions, which is highly variable [18], and potentially hampered by previously prioritising the role of clinicians as intervention deliverers to the detriment of harnessing their invaluable contribution in the design phase. Using the IM process to also understand and address the barriers to recruiting and retaining sufficient participants, the identification of sufficient numbers of suitable clinical sites, required adaptations to facilities, equipment and staffing and training requirements to support consistent intervention delivery across a range of primary care health settings enhanced our readiness to evaluate the intervention in the feasibility trial.

As demonstrated in this paper, the IM process details how accessing and using theory can be undertaken to support intervention development and implementation as highlighted in the MRC framework [20]. The application of this approach allows for meaningful analysis of the underlying mechanisms that are hypothesised to affect the desired intervention outcomes, by enabling the explicit linking of intervention components to theory, which should lead to improved outcomes for the targeted populations and an enhanced potential for intervention replication [28]. Our rapid review found that the majority of previous group-based SM interventions failed to report any underpinning behaviour change theory or techniques [40], reducing understanding of mechanisms of action, preventing replication and potentially contributing to their small effects [13, 75, 76]. This was compounded by the limited and variable quality of mediation studies for the target SM behaviours in OA and CLBP [48–51, 77] that required our pragmatic selection of behavioural determinants that could be targeted by the intervention. While self efficacy is an important determinant of physical activity in the general population and older adults with some evidence in OA and CLBP [48, 49], the more tenuous evidence for the effects of fear and catastrophizing [50, 51] on SM outcomes warrants further investigation in appropriately designed and powered prospective mediation studies [75]. Motivation was identified as a key determinant of SM behaviour and enhanced within the intervention by selecting SDT rather than other theoretical perspectives due its primary focus on an individual’s need for autonomy, a core component of SM. Other prominent psychological theories identified in our literature review [40, 75], such as social cognitive theory [78] (which was applied within FASA [35]), predominantly target constructs such as self efficacy, conceptually similar to competence within SDT [79], rather than autonomy. It was also considered unnecessary to include an additional behaviour change theory to target some of the other determinants, as SDT has been found to positively influence other mediators (i.e. fear) related to treatment [80], and the TDF provides a sound theoretical basis for targeting all our selected mediators. Furthermore, the evidence for the determinants of increasing participants’ SM knowledge and skills exemplified in our consolidated definition and highlighted in the physiotherapist focus groups was limited by their poor measurement in previous studies that should be addressed in future research [12, 81].

The study is limited by comparatively less engagement with people with OA and CLBP in the intervention development process that may have increased the acceptability and sustainability of the intervention, but will be addressed in the feasibility trial [36]. While it would have been preferable to specify the target behaviours in a more detailed way, most current OA and CLBP guidelines lack specificity in relation to physical activity and dietary changes for weight management [7, 82]. Indeed, recent evidence has reported health gains in those achieving below recommended physical activity levels [83, 84], and there is general consensus that due to concerns about pain exacerbation, people with chronic musculoskeletal pain should be supported to do activity according to their abilities [85, 86], as we have previously demonstrated in CLBP [87]. Nonetheless, the intervention included public health recommendations for 150 min of moderate intensity physical activity, as well as healthy eating and weight management guidance in addition to relevant statistics for the Irish population to promote behaviour change. While recommendations for resistance and flexibility exercises on 2 or 3 days each week [88] were conveyed to participants during SOLAS, they could have been specified more explicitly within the target behaviours without undermining autonomous motivation. In relation to the remaining SM behaviours, recent trials reporting positive effects have failed to quantify the use of pain coping skills, pharmacological or non-pharmacological pain management strategies by participants, thus limiting our ability to specify targets [89–91]. Within the feasibility trial, the proportion of participants achieving recommended levels of physical activity and using the SM behaviours will be explored to allow their specification for a future definitive trial. Finally, potential socio-cultural and environmental determinants of physical activity and diet in the general population were not specifically addressed within our intervention due to lack of evidence [92–94].

## Conclusions

This study provides a detailed example of the application of the IM approach to the development of a theory-driven, group-based complex intervention designed to promote self-management, for evaluation in a feasibility trial. While IM is a time-intensive collaborative process, the range of methods and resultant high level of transparency is invaluable and allows replication by future complex intervention and trial developers.

## Availability of supporting data

The data sets supporting the results of this article are included within the article and its additional files.

## Additional files

Additional file 1: Interview guides for semi-structured interviews and focus groups. (DOCX 71 kb)

Additional file 2: Review of reviews: defining chronic disease self-management [95–111]. (DOCX 43 kb)

Additional file 3: Primary care physiotherapy services resource capacity checklist results. (DOCX 18 kb)

Additional file 4: Overview of needs assessment results [112–114]. (DOCX 30 kb)

Additional file 5: Comparison of clinical guidelines for osteoarthritis and chronic low back pain to intervention prototype and adapted intervention [115–121]. (DOCX 37 kb)

Additional file 6: Results of feasibility analysis - focus groups. (DOCX 26 kb)

Additional file 7: Matrix of change objectives for self-management behaviour. (DOCX 19 kb)

Additional file 8: Matrix of change objectives for adoption and implementation. (DOCX 18 kb)

## Abbreviations

BCT: behaviour change technique
CLBP: chronic low back pain
FASA: Facilitating Activity and Self-management in Arthritis
GP: general practitioner
IM: intervention mapping
MRC: Medical Research Council
OA: osteoarthritis
PCCC: Primary, Community and Continuing Care
SDT: self-determination theory
SM: self-management
SOLAS: self-management of osteoarthritis and low back pain through activity and skills
TDF: Theoretical Domains Framework
TENS: transcutaneous electrical nerve stimulation
