# Supplementary material for: Using intervention mapping to develop a theory-driven, group-based complex intervention to support self-management of osteoarthritis and low back pain (SOLAS)
Source: Implement Sci. 2016 Apr 26;11:56. doi: 10.1186/s13012-016-0418-2 (PMC4845501; doi:10.1186/s13012-016-0418-2)
Supplement: Supplementary file 1 — Interview guides for semi-structured interviews and focus groups. (DOCX 71 kb) [file 13012_2016_418_MOESM1_ESM.docx]

**Additional file 1 Interview guides for semi-structured interviews and focus groups**

1. **Physiotherapy Manager Interview Guide**

**Introduction** (3 minutes)

Firstly, I would like to **thank you for taking the time** to meet with us today.

Have you read the information leaflet and informed consent form? Have you any questions? Before we start, I would like to remind you there are **no right or wrong answers** to the questions I will ask and you don’t have to answer any questions that you don’t feel comfortable talking about. If it’s ok with you, **the interview will be audio recorded** to ensure that all of your key points are accurately documented. Everything you say will be **kept confidential and anonymous**. You’re also welcome to request a copy of your interview transcript if you would like to review it for clarification, to add to it, or to indicate that all or part of it should not be used.

***[Request signature on informed consent form].***

***[Review service description form overleaf to check for completion, address any queries]***

[
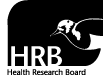
](http://www.hrb.ie/)
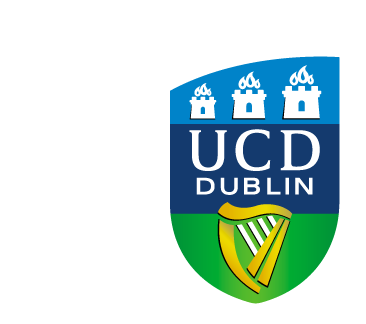


**Group education and exercise for self-management of clients with chronic musculoskeletal pain in primary care**

**Physiotherapy Service Description**

**Dear [NAME],**

We are scheduled to meet at **[TIME]** on **[DATE]** at **[LOCATION]** as part of the development phase of the Health Research Board funded study entitled ‘Group education and exercise for self-management of clients with chronic musculoskeletal pain in primary care using the Medical Research Council guidance for the evaluation of complex interventions’. In advance of this meeting, I would be very grateful if you could provide the information requested below and return to me as soon as possible. This information will help focus our interview to make the best use of our time together.

| 1. **Please confirm that your contact details are correct as follows:** | | **Is this correct?** | **If not, please specify:** |
| --- | --- | --- | --- |
| PCCC Area: | [PCCC Area] | Yes No |  |
| Telephone: | [TELEPHONE] | Yes No |  |
| Mobile: | [MOBILE] | Yes No |  |
| Email: | [EMAIL] | Yes No |  |
| Address: | [ADDRESS] | Yes No |  |

| 1. **How many physiotherapists in total currently provide services within your PCCC area:** | Number |
| --- | --- |
| On a full time basis? |  |
| On a part-time basis? |  |

| 1. **How many physiotherapists in total currently provide services for *clients with musculoskeletal conditions* within your PCCC area:** | Number |
| --- | --- |
| On a full time basis? |  |
| On a part-time basis? |  |

| 1. **Please estimate the number / percentage of the adult (18+ years) case-load within your PCCC area that the following conditions constituted in 2012:** | Number | % of total case-load |
| --- | --- | --- |
| Musculoskeletal conditions |  |  |
| Osteoarthritis of the lumbar spine or lower limb |  |  |
| Chronic low back pain (> 3 months duration) |  |  |

| 1. **Do you currently provide any group-based programmes for any of your clients?** | | Yes  No → Go to question 6 |
| --- | --- | --- |
| If yes, please specify which client groups these programmes are provided for and where these programmes are held: | | |
| Client group | Location of group programme | |
|  |  | |
|  |  | |
|  |  | |

| 1. **What are the main reasons that you are not currently providing any group-based programmes for any of your *clients with musculoskeletal conditions*?** | |
| --- | --- |
| Insufficient number of clients | Lack of appropriate facilities |
| Insufficient training/experience in running group classes | Lack of administrative support |
| Lack of interest from clients | Physiotherapy staffing issues |
| Others → please specify: | |

| 1. **Please provide the following information for each of the physiotherapists who currently provide services for *clients with musculoskeletal conditions* within your PCCC area:** | | | | | |
| --- | --- | --- | --- | --- | --- |
| Name | Email | Telephone | Grade | Site | Does this physiotherapist provide any group-based interventions for your clients? |
|  |  |  |  |  | Yes No |
|  |  |  |  |  | Yes No |
|  |  |  |  |  | Yes No |
|  |  |  |  |  | Yes No |
|  |  |  |  |  | Yes No |
|  |  |  |  |  | Yes No |
|  |  |  |  |  | Yes No |

**Thank you for your time, we look forward to meeting with you to learn more about your physiotherapy service. If you have any questions, please contact the Project Manager Dr Laura Currie-Murphy at 01 716 6523 or by email at** [**laura.currie-murphy@ucd.ie**](mailto:laura.currie-murphy@ucd.ie) **or the Principal Investigator Dr Deirdre Hurley-Osing at 01 716 6524 or by email at** [**deirdre.hurleyosing@ucd.ie**](mailto:deirdre.hurleyosing@ucd.ie)**.**

**Management of Chronic MSK Pain** (4 minutes)

What are some of the **biggest challenges your service** is currently facing?

We are particularly interested in your client population with Osteoarthritis of the lumbar spine, hip or knee and chronic low back pain – with duration of > 3 months.

***[Refer to service description questionnaire for caseload burden of musculoskeletal conditions, osteoarthritis and chronic low back pain].***

To what extent is **managing chronic musculoskeletal conditions,** such as osteoarthritis and low back pain, **a priority within your service**? **G**

**How does your team currently manage clients** with chronic musculoskeletal conditions such as osteoarthritis and chronic low back pain? **S**

- Can you describe any **successes or challenges you have in providing services** for clients with these conditions? **S, R, B**
- Are there **any interventions that are not currently provided** that you think need to be provided for clients with chronic musculoskeletal pain to improve outcomes for them?

**Group-based programmes** (12 minutes)

***Questions for Primary Care Areas currently providing group-based programmes***

*[Refer to question 5 of the service description questionnaire to see what groups they are currently running. Focus questions specific to groups run for clients with chronic musculoskeletal pain if there are multiple groups].*

**Can you briefly describe the group-based programmes *[specify which one if applicable]* that are run in your area?**

- What **types of clients** are they for?
- What is the **purpose** of these programmes?
- What does the **education component** include?
- Is there a **focus on promoting self-management** or encouraging clients to take an active role in managing their condition and if so how?
- Was there any attention paid to what clients did outside of the class to manage their condition? How was this addressed?
- **How** **often** are the groups run? (Weekly, biweekly, number of classes, number of times per year etc)
- **When** are they run?
- **Where** are they run?
- **Who** runs them?
  - What skills do you feel are important for those who are running groups to have?
  - Are there any skills that you feel need further development?
- **Who organises them**? Do you have **administrative support** to help organise groups?
- How are **clients identified** for these groups?
  - How do you **refer** into the groups? Assessed first then group? Or do they receive individual treatment before entering a group?
  - Are there any types of clients that you feel would be **inappropriate for groups**?
  - Have you had any **safety concerns** or issues in groups? How are these prevented or handled?
- What **role** (if any) does the **wider primary care team** play in these group programmes?
- What are some of the **successes or challenges** you have faced in introducing/running these groups?
- What are some of the **advantages/disadvantages of group versus individual** treatment programmes?
  - From a service delivery or organisational perspective?
  - In terms of client outcomes?
  - In terms of quality of care?
- In your opinion, what are the **key things that are required to run a successful group**-based programme in your area?
  - (e.g., appropriate facilities, adequate admin support, “bought in” physiotherapists)

**Group-based programmes** (12 minutes)

***Questions for Primary Care Areas currently not providing group-based programmes***

**You indicated that there are currently no group-based physiotherapy programmes in your area. Have you any past experience of group-based programmes within this service area or another primary care setting?** *[If no past experience, skip to next question]*

- Can you **describe** this past experience?
- What **types of clients** were they for?
- What was the **purpose** of these programmes?
- What did the **education component** include? How was this delivered?
- Was there a focus on **promoting self-management** or encouraging clients to take an active role in managing their condition if so how?
- Was there any attention paid to what clients did outside of the class to manage their condition? How was this addressed?
- How **often** were the groups run? (Weekly, biweekly, number of classes, number of times per year etc)
- **When** were they run?
- **Where** were they run?
- **Who** ran them?
- **Who organised them**? Did you have **administrative support** to help organise groups?
- How were **clients identified** for these groups?
- What **role** (if any) did the **wider primary care team** play in these group programmes?
- What were some of the **successes or challenges** you faced in introducing/running these groups?
- What were some of the **advantages/disadvantages of group versus individual** treatment programmes from an organisational perspective?
- **Why** were these groups **discontinued**?

*[Refer to Question 6 of the service description questionnaire]* **You indicated that the main reasons you are not currently providing any group-based programmes were…**

- Can you tell me a little bit more about this?

**Are you considering introducing group-based programmes for people with chronic musculoskeletal pain in the future?**

- **Why? / Why not?**
- Would you be **interested in being involved** in the development of a group-based programme within the context of this project?
- In your opinion, what are the **key things that would be required to run a successful group** based programme in your primary care area?
- What **skills** do you feel are **important for those who are running groups** to have?
  - Are there any **skills** that you feel need **further development** within your team to successfully provide groups?
- What are some of the **successes or challenges** you would expect to face in introducing these groups?
- What are some of the **advantages or disadvantages** of group versus individual treatment programmes from an organisational perspective?

**Intervention components** (5 minutes)

The aim of our study is to develop a group-based programme to promote self-management of clients with osteoarthritis of the lumbar spine of the lower limbs and chronic low back pain. We are currently reviewing the evidence from the literature and existing programmes that are being run locally and internationally to inform this intervention and we would be interested in your views on how a group-based exercise and education programme to promote self-management for clients with chronic pain might actually work within your primary care area.

- Is there anything that you think would **help or hinder the implementation** of such an approach within your area?
- What are your thoughts about the **length of the programme**? **How many sessions** should it include and how **frequent** do you think would be practical and feasible?
- What are your views on a **mixed class** incorporating clients with **chronic low back pain and OA**? Would this be feasible? Do you foresee any challenges?
- **Where** would you foresee such a programme being run within your area?
  - How many people could be accommodated in a class in this facility?
- What **exercise equipment** do you have available in this facility?
  - Would you have access to equipment such as stationary bikes, cross trainers, treadmills, gym balls, rowers, steppers, etc.
- Is there **anything that you would need** to enable you to provide such a programme?
- Do you think such a programme would be **well received by your clients**?
- Do you think such a programme would be **well received by the wider primary care team** in your area?
- What role (if any) would you foresee the wider primary care team playing in providing such a programme?

**Self-management** (3 minutes)

Are you familiar with self-management approaches to chronic musculoskeletal pain conditions?

- Do you **promote self-management approaches for any conditions** within your service?
- Have you any **experience of providing self-management** approaches for people with chronic musculoskeletal pain conditions?
- What would you **expect a self-management approach** for people with chronic musculoskeletal pain conditions such as chronic low back pain and **osteoarthritis to consist of**?
- What would you see as some of the **advantages and disadvantages of promoting self-management** among your clients with osteoarthritis and low back pain?

**Information about the study – next steps** (3 minutes)

***Physiotherapy focus group interviews***

Between August and October, we would like to interview some of the physiotherapists in your area to learn from them about their experience of treating people with chronic MSK pain, understand the context in which they are working, and identify any barriers or enablers that they foresee would help or hinder the implementation of a group-based exercise and education intervention to promote self-management among their clients with chronic MSK pain.

*[Refer to Question 7 of service description questionnaire to see who from their team provides group and individual interventions]*

**Is there anyone within your service who works in the musculoskeletal area that is currently providing groups/interested in providing groups that you think would be interested in meeting with us?**

***Client interviews***

We may also be interested in interviewing a sample of your clients between August and December to learn from them their expectations of physiotherapy services, their experiences of self-management behaviours, and to identify any barriers or enablers that they foresee would help or hinder their participation in a group-based exercise and education programme.

To facilitate these interviews, we would be grateful if a physiotherapist from within your service could provide an information leaflet to eligible clients – these will be people aged 18 years and over with OA or chronic low back pain who have accessed your service in the past six months or enter the service between September and November. If the clients are interested and willing to participate they can contact us directly to schedule the interview at a convenient time and location.

***Symposium***

Lastly, in October we will be holding a symposium at UCD. We will be presenting the findings of these interviews with the Managers and Physiotherapists. In conjunction with this symposium we will be holding a series of workshops where you will be provided an opportunity to input and shape the development of the group exercise and education programme for the current project.

The symposium is supported by the HRB and is free of charge. It would be a great free CPD opportunity for your physiotherapists. Do you think that you and some of your physiotherapists will be able to attend this symposium? How many people do you think we could expect to attend from your area?

**Close of interview**

**Do you have any questions about the study? You’re welcome to contact me at any time if you have any further questions. Thank you again for your time and we look forward to working with you.**

1. **Patient Interview Guide:**

**Introduction**:

Thank you for agreeing to speak with me today about how you cope with your condition. It is important to note that there are no right or wrong answers to the questions and anything you say will be stored anonymously for the purpose of the research. This interview should take about **15-20 minutes and** will be audio-recorded to ensure that all of your key points are accurately documented. You do not have to answer or disclose anything that you do not want to, and you are free to end the interview or withdraw from the study at any point.

I’d like to now just take a moment for you to review the information leaflet again, and if you have any further questions please ask them.

**Note: At this point, participant will be asked to sign consent form if not already completed.*

**Background data:**

Male/Female: _____________________________

Age Range: 18-25 26-35 36-45 46-55 56-65 66-70

Diagnosis/es:

____________________________________________________________________________

Length of time since diagnosis/es (years):

____________________________________________________________________________

Any other health problems/medical history:

____________________________________________________________________________

Brief record of 0065xperience of physiotherapy to date:

____________________________________________________________________________

**EXPERIENCE of GROUP PHYSIOTHERAPY:**

- What was your previous experience of group physiotherapy like? *E.g. by group physiotherapy we mean classes or sessions led or facilitated by a physiotherapist involving more than one person (or patient).*
  - Who took part in it *(e.g. just LBP/OA or mixed CMP?)*
  - Did you enjoy it? Why? Why not? (E)
  - What did you think might have improved your experience?
  - Did you attend all the sessions? Why not?

**In particular, what kinds of things would you think influence you taking part in a *group* physiotherapy programme to help you take control and manage your own condition? What might put you off? What might encourage you to take part?.............**

*Prompts:*

- Anything in particular, like what we just discussed?
  - *Like occupation, how you think or feel about your condition, the people around you, practical issues etc*
- What would you like to get from a group physiotherapy programme that aimed to help you manage your LBP/OA? (I)
  - E.g. Do you think more education about self-management strategies would be useful? What kinds of things would you like them to educate you on?
    - *E.g. appropriate physical activity, knowledge about your condition, advice on pain management, goal-setting etc*
      - *Would you prefer goal-setting to be done with you, and reviewed at every session? Or would you prefer to be shown how to do it, and then left to your own devices as to whether you use it or not?*
  - How would you like this delivered?
    - *E.g. group discussion, teacher-pupil style, workbooks, diaries etc*
  - What would your feelings be on the group consisting of people with differing conditions, like low back pain and osteoarthritis?
  - What would your feelings be on the group consisting of different age-groups?
- What type of setting for physiotherapy would you prefer ideally if you were to use it to help you cope? Why? E.g. (I)
  - *group v individual*
  - *clinic v home*
  - *duration of sessions*
    - *How long of a session could you attend?*
    - *Would you be able to attend twice a week?*
    - *Would you be able to attend during the day?*
  - What types of things influence these preferences?

**Conclusion:**

Thanks very much for taking part. I think we have covered most of what we wanted to talk about. Is there anything else you would like to add before we finish up?

1. **Focus groups with PCCC Physiotherapists Interview Guide**

**Objectives**

The objectives of the focus group are as follows:

- To assess attitudes towards group exercise, education, and self-management components of the FASA intervention ([Walsh et al., 2012](#_ENREF_109)) to determine their feasibility and acceptability; and
- To identify understanding/perceptions, barriers and enablers to providing a group-based education and exercise programme to promote self-management for people with chronic musculoskeletal pain from clinician and client perspectives.
- To identify potential clinician-related barriers to the implementation fidelity of this programme

**Programme**

Part A: Overview and Introductions (30 minutes)

Part B: Intervention structure and content (55 minutes)

Part C: Summary and next steps (10 minutes)

**Part A: Introduction** (approx. 30 mins)

1. **Refreshments** (10 mins)
2. **Overview** (approx. 2 minutes)

- Brief explanation of the purpose of the overall study and focus group (DH)
- Overview of the structure of the focus group (SG)
- Explanation of ground rules (SG)
  - Recording for transcription
  - One person speaking at a time
  - Confidentiality
  - Consent

1. **Introductions** (approx. 8 minutes) (SG)

- Study team
- Name and past or current experience of group-based physiotherapy for chronic MSK pain.
  - Do you have any past or current experience of providing group-based programmes for clients with chronic MSK pain?

1. **Discussion** (approx. 10 mins) (SG)

- What is your general experience and views of group-based approaches to physiotherapy for chronic musculoskeletal conditions?
  - Successes, challenges
  - How did you motivate clients to attend, stay in the group?

**Part B: Intervention Structure and Content** (approx. 55 mins)

1. **Presentation of Structure & Content of the FASA intervention** (approx. 12 mins) (DH)

- Aim of the intervention
- Brief overview of evidence-base underpinning intervention
- Population – OA of hip knee and lumbar spine, chronic low back pain
- Structure
  - Group-based – delivered to groups of approximately 8 participants
  - Health professional led
  - 6 week, twice weekly
- 15 – 20 minutes of physiotherapist-led group discussion and problem solving session with supporting hand outs regarding issues of self-management
  - Topics include: activity-rest cycling, use of ice and heat for pain relief, goal-setting and action plans, exercise recommendations and healthy eating and managing changes in pain.
- 35 to 40 minutes of exercise, based on a circuit of strengthening, aerobic and co-ordination activities.
- Physiotherapist and participant collaboratively complete an action plan regarding exercise/activities the participant wants to achieve over the following week. This will be reviewed after each week, to determine adherence to the plan, problem-solving if the goal has proved unachievable, or progressed if achieved.
- Intervention will be underpinned by behaviour change theory and will employ specified behaviour change techniques
- Any specific questions for clarification?

1. **Discussion Topics (approx. 43 mins)**  (SG)

**HIGH PRIORITY**

- What are your initial impressions about the structure and content of the intervention? (4 mins)
- What do you think about the feasibility of this intervention within your service delivery context? [Capability, Opportunity] (10 mins)
- Is there any (other) specific feedback you have on the feasibility of implementing this intervention in your service setting with these client groups? [Capability, Opportunity] (10 mins)
  - - Population – sufficient number of appropriate clients, combining clients with different pain sites - OA hip, knee and back and CLBP ECR
    - Structure – 6 weeks, twice weekly, balance of education (15-20 mins) and exercise (35 – 40)
    - Knowledge / Skills to facilitate group, provide educational component K S BCap
    - Facilities and resources (appropriate facilities, equipment, staffing) ECR
    - Managerial support (service managers, referral sources, general practitioners and multidisciplinary team) SI SPRI
  - Of all of the challenges you have mentioned, which ones are the biggest or most important? How might the most important challenges be addressed?

**LOWER PRIORITY**

- What resources or support do you feel you would require to successfully provide this intervention? (note responses on flip chart) [Capability, Opportunity] (8 mins)
  - - Is there anything you would need to feel confident providing education in a group format and covering the topics specified (put up slide with the topics listed: activity-rest cycling, use of ice and heat for pain relief, goal-setting and action plans, exercise recommendations and healthy eating and managing changes in pain) K S BCap E BR
    - Is there anything you would need to feel confident providing a circuit-based structured exercise programme to a group with OA and chronic low back pain? (ie training, a second facilitator for support) K S BCap E BR
    - Two physios or physio + assistant
  - Which, if any of these resources/supports, are absolutely essential – for example you could not provide this intervention without them?

**Part C: Summary and next steps** (10 minutes)

**One slide summarising next steps** (LM)

- Analysis of focus groups – modification of the intervention based on feedback from Managers Interviews and focus group
- Symposium in October – , presentation of the intervention, consensus on intervention, sign up, research readiness
- Trial to be conducted in waves prioritising those who are most ‘research ready’

1. **Discussion**

So our aim is to go away from here and adapt the intervention based on your feedback to ensure that it is acceptable to you and feasible to be implemented within the settings where you are working. To finish off, if you were asked to deliver this intervention next week, what is the one thing you would need us to address/change/provide between now and then? [go around to each and ask for a response] * Note slide at end with contact details – any other issues ideas that arise – they are welcome to contact team and these ideas will be considered.

**Coding**

| **Acronym** | **Theoretical Domains Framework [39], [44]** |
| --- | --- |
| K | Knowledge |
| S | Physical skills |
| BCap | Beliefs about capabilities |
| BCon | Beliefs about consequences |
| SI | Social Influences |
| E | Emotions |
| O | Optimism |
| I | Intentions |
| MAD | Memory, attention and decision making processes |
| ECR | Environmental context and resources |
| SPRI | Social/Professional role and identity |
| BR | Behavioural regulation |
| G | Goals |
| R | Reinforcement |

| **Intervention Components** | | **Experiences & Attitudes** | |
| --- | --- | --- | --- |
|  |  | **Negative** | **Positive** |
| ***Programme Participants*** | - Number of Suitable Participants - Mixed Group (OA, LBP, age) | Part-  Mixed- | Part+  Mixed+ |
| ***Programme Content*** | - Circuit-Based Exercise - Education - Self-Management | Circuit-  Education-  SM- | Circuit+  Education+  SM+ |
| ***Programme Structure*** | - Group-based - 6 Weeks - Twice Weekly - 1-hour Classes | Group-  6 Weeks-  Twice-  1H- | Group+  6 Weeks+  Twice+  1H+ |
| ***Programme Delivery*** | - Staffing (no. of physiotherapists, MDT involvement, admin) - Facilities/Equipment - Managerial Support - Referrer Support | Staff-  Facilities-  Manage-  Referrer- | Staff+  Facilities+  Manage+  Referrer+ |

**Coding Framework for Feasibility Analysis**

**Coding Framework for Barriers & Enablers Analysis**

| **COM-B Components** | | **TDF Domains** | **Barrier** | **Enabler** |
| --- | --- | --- | --- | --- |
| 1. Capability | 1.1 Psychological | 1.1a Knowledge  1.1bCognitive & interpersonal skills  1.1c Memory, attention and decision processes  1.1d Behavioural regulation | 1.1a-  1.1b-  1.1c-  1.1d- | 1.1a+  1.1b+  1.1c+  1.1d+ |
|  | 1.2 Physical | 1.2 Skills | 1.2- | 1.2+ |
| 1. Opportunity | 2.1 Social | 2.1 Social influences | 2.1- | 2.1+ |
|  | 2.2 Physical | 2.2 Environmental context and resources | 2.2- | 2.2+ |
| 1. Motivation | 3.1 Reflective | 3.1a Professional/social role and identity  3.1b Beliefs about capabilities  3.1c Optimism  3.1d Beliefs about consequences  3.1e Intentions  3.1f Goals | 3.1a-  3.1b-  3.1c-  3.1d-  3.1e-  3.1f- | 3.1a+  3.1b+  3.1c+  3.1d+  3.1e+  3.1f+ |
|  | 3.2 Automatic | 3.2a Reinforcement  3.2b Emotion | 3.2a-  3.2b- | 3.2a+  3.2b+ |
