# Supplementary material for: Using intervention mapping to develop a theory-driven, group-based complex intervention to support self-management of osteoarthritis and low back pain (SOLAS)
Source: Implement Sci. 2016 Apr 26;11:56. doi: 10.1186/s13012-016-0418-2 (PMC4845501; doi:10.1186/s13012-016-0418-2)
Supplement: Supplementary file 2 — Review of reviews: defining chronic disease self-management [95–111]. (DOCX 43 kb) [file 13012_2016_418_MOESM2_ESM.docx]

**Additional file 2 Review of reviews: defining chronic disease self-management**

Method: ‘self-management’ search terms were combined with ‘review’ search terms, publication lists of below databases were searched.

| **Study (reference)** | **Definition of ‘Self-Management’** | **Search Period** | **Databases searched** | **Inclusion** | **Search terms** |
| --- | --- | --- | --- | --- | --- |
| **Process** | | | | | |
| Warsi et al. [95] | Self-management programs facilitate  acquisition by the patient of preventive  or therapeutic health care activities,  often in collaboration with health care providers. Self-management education programs emphasize the role of patient education in preventive and therapeutic health care activities and usually consist of organized learning experiences designed to facilitate adoption of health-promoting behaviors. | January 1, 1964, through January  31, 1999 | MEDLINE and HealthSTAR | Controlled studies, some randomised, some not. | Self-management,  self-care, demand management, patient education,  self-efficacy, social learning theory, arthritis, osteoarthritis,  rheumatoid arthritis, diabetes, hypertension, asthma,  hypertension, congestive heart failure, and chronic disease |
| Chodosh et al. [96] | Chronic disease self-management is a  systematic intervention that is targeted toward patients with chronic disease. The intervention should help them actively participate in either or both of the following: self-monitoring (of symptoms or of physiologic processes) or decision making (managing the disease or its impact  through self-monitoring). | From 1980 to September 2004 | An Indexed Bibliography on Self-Management for People with Chronic Disease, published by the Center for Advancement of Health, MEDLINE, PsycINFO, and CINAHL, Cochrane Library, Assessment of Self-Care Manual | RCTs, reviews | Arthritis, Chronic Illness, Diabetes, Heart Disease, and  Hypertension. Keywords used in combination with disease names included Anxiety, Behavior, Compliance, Coping , Depression , Disability, Exercise, Family, Isolation, Modeling, Nutrition, Patient Education, Patient Satisfaction, Problem-Solving, Relaxation, Self-care ,and Social Support |
| Devos-Comby et al. [97] | Self-management interventions were defined as programs focusing on education about OA, OA self-management or self-care (for example, the Arthritis Self-Help Course), and pain coping skills, as well as diet self-management | January 1966 to May  2005 | Medline and PsycInfo | RCT or quasiRCT | “osteoarthritis” and “knee,”  with “intervention,” “education,” “exercise,” or “self-care |
| Walsh et al. [98] | No definition reported.  Content of the self-management component was frequently ill-defined, but generally included ‘advice and education on osteoarthritis’, healthy life-styles, exercise adherence strategies, and pain-management  techniques. | From inception to December  2005 | Medline,  EMBASE, AMED, CINAHL and PsycInfo. Cochrane Library, National Research Register, DARE, PEDRO, NICE and HTA databases | RCTs | ‘osteoarthritis/chronic joint  pain’ were combined with ‘exercise’ and ‘self-management’. Terms were also exploded |
| Zwar et al. [99] | Weingarten’s definition of chronic disease management is “an intervention designed to  manage or prevent a chronic condition using a systematic approach to care and  potentially employing multiple treatment modalities”  Self-management support: Collaboratively helping patients and their families to acquire the skills and confidence to manage their condition. Provide self management tools, referrals to community resources, routinely assessing progress | January 1990 to February 2006 | Medline, Embase, CINAHL and PsychLit  and the Cochrane Effective Practice and Organisation of Care (EPOC) specialised  register | RCTs, CCTs (clinical controlled trials), CBA (controlled before and after), ITS (interrupted time series studies) | Terms for the chronic diseases of interest and aspects of  chronic disease management were combined with terms for primary and community  care |
| Foster et al. [100] | Self-management education programmes defined as structured programmes for people with chronic conditions involving an iterative process of interaction between the participant and the tutor | Earliest available – July 2006 | CENTRAL, MEDLINE, EMBASE, AMED, CINAHL, DATE, PsychINFO, NHS Economic evaluations database, Science Citation Index, | RCTs of self-management programmes for people with chronic conditions |  |
| Reid et al. [101] | Self-management programs differ with respect to target audience, mode of delivery (group vs individual) and intervention components; however, share common features: employ strategies to reduce pain by altering behavioural, cognitive or emotional responses and enhance self-efficacy for managing pain.  Programmes typically include some combination of: education about pain and its consequences, relaxation skills training, cognitive coping skills training, problem solving, and communication skills training. | 1980 - 2007 | MEDLINE, Cumulative Index to Nursing and Allied Health | Self-management interventions for chronic musculoskeletal disorders among older people | Self-care, patient education, arthritis, osteoarthritis, pain, yoga, massage and Tai Chi, aged, chronic pain, self-management, Arthritis Foundation |
| Nunez et al. [102] | Self-management programs differ from health education  in that they are designed to improve the self-management of the condition in individuals with the disease. Established programs are multicomponent and incorporate information about chronic disease, an overview of self-management principles, exercise, cognitive symptom  management, dealing with difficult emotions, nutrition, communication with family and health care providers, and behavioral contracting. This type of intervention aims to enhance perceived ability to control various aspects of  disease by improving self-efficacy through skill mastery, modeling, persuasive communication, and reinterpretation of symptoms. | From 1985 to 2008 | MEDLINE, CINAHL, and the Cochrane Library | RCT or quasiexperimental | “adult,” “older  adult,” “arthritis,” “rheumatoid arthritis,” “fibromyalgia,”  “stiffness,” “standard care,” “primary care,” “functional  health,” “functional disability,” “disability prevention,”  “quality of life,” “physical mobility,” “disease management,” “self-care,” “self-management,” “self-efficacy,” “social isolation,” “fatigue,” “pain,” “depression,” and “cost-effectiveness.” |
| Miles et al. [49] | Self-management programmes are defined as multi-component interventions directed at patients, aimed at principally improving participants’ health status or quality of life where there is scope for improvement in patients’ managing their own health, with a taught or self-taught component aimed at increasing participants’ skills and knowledge and enabling participants to deploy these enhanced skills in aspects of their lives beyond the intervention. Psychological components had to be behavioural and/or cognitive and/or structured (not psychodynamic therapy). | 1984 – April 2009 | MEDLINE, EMBASE, PsychINFO, CINAHL, AMED, Web of Science, Cochrane Library | RCTs of self-management programmes for chronic musculoskeletal pain | chronic musculoskeletal pain, back pain, neck pain, shoulder pain, knee pain, hip pain, fibromyalgia, osteoarthritis  self-management, self-care, self-efficacy, self-help, self-improvement, patient education, patient teaching, patient training, expert patient, lay-led, peer-led, professionally-led |
| Carnes et al. [19] | Self-management program defined as a multi-component, structured, taught, or self-taught course with distinct components, principally aimed at patients with the goal of improving participants’ health status or quality of life by teaching them skills to apply to everyday situations.  Contain at least 2 components from the following 5 groups: psychological (including behavioural or cognitive therapy), mind-body therapies (including relaxation, meditation, or guided imagery), physical activity (including any form of exercise), lifestyle (such as dietary advice or sleep management), and pain education (such as understanding their condition or how to take medication effectively). | January 1994 – April 2009 | MEDLINE, EMBASE, PsychINFO, CINAHL, AMED, Social Sciences Citation Index, Cochrane Library | RCTs of chronic musculoskeletal pain in adults 18+ years. Chronic pain defined as lasting longer than 3 months. | Chronic musculoskeletal pain, back pain, neck pain, shoulder pain, knee pain, hip pain, fibromyalgia, osteoarthritis  Self-management, self-care, self-efficacy, self-help, self-improvement, patient education, patient teaching, patient training, expert patient, lay-led, peer-led, professionally-led |
| Kroon et al. [13] | Self-management programmes are structured education programmes judged as being primarily educational and address self-management of osteoarthritis. Programme components that directly address self-management may include fostering skills in managing OA, such as problem solving, goal setting, decision making, self-monitoring, coping with the condition, or interventions to manage pain or improve physical and psychological functioning |  |  |  |  |
| New Zealand Guidelines Group [103] | Some definitions of chronic disease  self-management include:  ‘…promotes the adoption of skills, behaviours and coping strategies to  enable patients to actively participate in their health care and decision  making and to maintain health and well-being.’  ‘The systematic provision of education and supportive interventions by  health care staff to increase patients’ skills and confidence in managing  health problems, including regular assessment of progress and problems,  goal setting and problem solving support.’  Must be evidence of **multiple** components that may include goal setting, self-efficacy,  self-monitoring, treatment adherence, as well as an educational component (ie. knowledge, skills and confidence) to be considered self-management | From 2005 to the  present | Medline, Embase, PubMed, Cinahl, all EBM resources on Ovid – this includes the Cochrane Database, ERIC (Education), PsychInfo, Web of Science, CCTR, Sociological Abstracts, APAIS. Guidelines International Network, Ministry of Health, Google, National Guideline Clearing House, TRiP Database, National Electronic Library for Health | Guidelines, SRs, RCTs of health behaviour change interventions (self-management interventions) | Too long to include |
| Nolte and Osborne [71] | If studies did not directly refer to Stanford SM, studies were selected that evaluated interventions that included at least two of the three keywords ‘problem-solving’, ‘action planning’, and ‘relaxation’. To be included in the review, four characteristics had to be met by the self-management  program:  a. Interventions were delivered in a group setting;  b. Interventions were based on a formal syllabus;  c. Interventions ran between four and ten sessions  within a period of 3 months;  d. Interventions did not include any additional components such as exercise lessons, reinforcement  techniques, individual consultations, and/or home  visits. | studies between 1982 and 2006 | MEDLINE, EMBASE, CINAHL, and PsycINFO | RCTs | patient education’ or ‘self-management’) and (‘randomised’ or ‘randomized’ or ‘RCT’) and (‘arthritis’ or ‘asthma’ or ‘chronic condition’ or ‘chronic disease’ or ‘chronic  obstructive pulmonary disease’ or ‘congestive heart failure’ or ‘COPD’ or ‘diabetes’ or ‘ﬁbromyalgia’ or ‘hypertension’ or ‘musculoskeletal’ or ‘osteoarthritis’ or ‘osteoporosis’ or ‘pain’ or ‘rheumatoid’ or ‘stress’ |
| Oliveira et al. [76] | This review employed the definition of self-management from the Australian First National Primary Health Care Strategy. Self-management is defined as a model of care which incorporates the following 6 core components: 1) knowledge of the condition and management options, 2) shared responsibility for a plan of care, 3) participation in health-promoting activities, 4) self-monitoring and management of signs and symptoms, 5) management of impact on physical functioning and emotional and personal relationships, and 6) confidence in use of support services. | Earliest record to April 2011 | MEDLINE, EMBASE, SINAHL, PsychINFO, LILACS, PEDro, AMED, SPORTDiscus, Cochrane Library | RCTs of self-management interventions for nonspecific low back pain of any duration | Cochrane collaboration back review group for clinical trials of LBP and “self-management” and “self-care” |
| **Behaviours/Outcome** | | | | | |
| Clark et al. [104] | Self-management has, in the main, been discussed as the day-to-day tasks an individual must undertake to control or reduce the impact of disease on physical health status. At-home management tasks and strategies are undertaken with the collaboration and guidance of the individual’s physician and other health-care providers. Self-management also requires the ability to cope with psychosocial problems generated or exacerbated by chronic disease. Successful self-management requires mastery of three separate but related categories of activities: knowledge about condition and treatment to make informed decisions about care, perform activities aimed at management of the condition, apply skills to maintain psychosocial functioning. | Not specified | Not specified | Not specified | Not specified |
| Lorig and Holman [105] | Self-management involves 3 tasks: medical management, emotional management, and social/role management  Self-management involves 6 skills: problem solving, decision-making, resource utilisation, forming a patient-provider relationship, taking action to resume activities. |  |  |  |  |
| Barlow et al. [106] | Self-management refers to the individual’s ability to manage the symptoms, treatment, physical and psychosocial consequences and life style changes inherent in living with a chronic condition. Efficacious self-management encompasses ability to monitor one’s condition and to effect the cognitive, behavioural, and emotional responses necessary to maintain a satisfactory quality of life. Thus a dynamic and continuous process of self-regulation is established. | Not stated. | BIOMED, BIDS, CINAHL, MEDLINE, PSYCHLIT, Cochrane Library | Reports of self-management approaches or evaluations of self-management interventions for chronic conditions broadly (predominately asthma, diabetes, and arthritis) | Self-management and chronic |
| Clark [107] | Self-management is defined as ‘day-to-day tasks an individual must undertake to control or reduce the impact of disease on physical health status. At-home management tasks and strategies are undertaken with the collaboration and guidance of the individual’s physician or other healthcare providers’  Successful self-management of chronic conditions requires sufficient knowledge of the condition and its treatment, performance of condition management activities, and application of the necessary skills to maintain adequate psycho-social functioning. |  |  |  |  |
| Johnston et al. [108]  *Definition same as [106] | Self-management refers to an individual’s ability to manage the symptoms, treatment,  physical, psychosocial, and lifestyle changes inherent in living with a chronic condition.  Self-management programmes seek to empower individuals to cope with disease and live better quality lives with fewer restrictions from their illness by developing self-efficacy, which is the level of confidence that an individual has in his or her ability to succeed in dealing with their own chronic disease.  Refers to the measures that a patient can take to manage and prevent the symptoms of his or her chronic disease in accordance with the participation of his or her health care team. Patient self-management necessarily requires the culmination of a certain set of skills and behaviors that create confidence in dealing with a range of emotional, physical and physiological symptoms of their chronic disease. Physician self-management support requires performing a certain set of tasks to guide the patient towards empowerment by encouraging and engaging the patient in their own management process. | 2006 to February 2008 | Medline, CINAHL and EMBASE | SRs, Meta-analyses, RCTs and qualitative studies | self-management, self-care, self-administration, self-help, self-efficacy, patient education, patient compliance, patient participation, chronic disease, chronic illness care, chronically ill, chronic condition, long term care; cancer, diabetes mellitus, heart disease, hypertension, stroke, asthma, chronic obstructive pulmonary disease, arthritis; primary health care, family physician, family health care, community health services; and systematic reviews, meta-analysis, randomized controlled trials (RCT) and qualitative studies |
| Coster and Norman [109] | Self-management has been defined as ‘the individual’s ability to manage the symptoms, treatment, physical and psychosocial consequences and lifestyle changes inherent in living with a long term disorder’ (DoH) [8]  ‘self-care’ is defined by the World Health Organisation (WHO) as including ‘activities that individuals, families, and communities undertake with the intention of enhancing health, preventing disease, limiting illness, and restoring health’ (WHO [95] | From inception to 2007 | Cochrane database 2007 | Cochrane SRs | educat*” “self-management,” and “self-care   “Patient education” and “Self-care” |
| May [110] | Self-management is related to the management of the day-to-day impact of a condition by the individual with that condition and include the following key elements: health promotion activities, self-monitoring of status, forming a patient-clinician relationship, problem solving, decision-making, and resource utilization. | Earliest available to April 2009 | MEDLINE, AMED, CINAHL, PSYCHLIT, Cochrane library | RCTs of self-management interventions for chronic low back pain and osteoarthritis | Self-management, chronic disease, pain |
| Du et al. [75] | Employed Barlow’s definition: ‘the individual’s ability to manage the symptoms, treatment, physical and psychological consequences and lifestyle changes inherent in living with a chronic condition’.  Self-management distinguishes itself from traditional patient education by emphasising the following process elements:   1. Self-efficacy building 2. Self-monitoring 3. Goal-setting and action-planning 4. Decision-making 5. Problem-solving 6. Self-tailoring 7. Client-clinician partnership | 1970 – March 2010 | MEDLINE, EMBASE | RCTs of self-management interventions for people with chronic musculoskeletal pain | Chronic musculoskeletal pain, arthritis, osteoarthritis, rheumatoid arthritis, fibromyalgia, back pain, neck pain, shoulder pain  Self-management, self-care, patient education  Randomized controlled trial or random |
| Jang and Yoo [111] | Self-management refers to an individual’s activities and means for controlling and caring his/her own disease [91] | January 2000 to  September 2009 | Korea Research Information Center  for Health, Korea Education and Research Information Service, Korea Library of Congress, Medline, Cochrane Library, and Cumulative  Index to Nursing and Allied Health Literature | Experimental or quasi-experimental design | Not given |
