# Supplementary material for: Using intervention mapping to develop a theory-driven, group-based complex intervention to support self-management of osteoarthritis and low back pain (SOLAS)
Source: Implement Sci. 2016 Apr 26;11:56. doi: 10.1186/s13012-016-0418-2 (PMC4845501; doi:10.1186/s13012-016-0418-2)
Supplement: Supplementary file 3 — Primary care physiotherapy services resource capacity checklist results. (DOCX 18 kb) [file 13012_2016_418_MOESM3_ESM.docx]

**Additional file 3 Primary care physiotherapy services resource capacity checklist results**

| **Site ID** | **Equipment %** | **Facilities %** | **Staffing %** | **Weighted total** |
| --- | --- | --- | --- | --- |
| **A** | 47.6 | 78.6 | 100.0 | 85.1 |
| **B** | 42.9 | 78.6 | 100.0 | 84.6 |
| **C** | 85.7 | 85.7 | 100.0 | 92.1 |
| **D** | 52.4 | 78.6 | 100.0 | 85.6 |
| **E** | 47.6 | 78.6 | 100.0 | 85.1 |
| **F** | 23.8 | 78.6 | 100.0 | 82.7 |
| **G** | 42.9 | 85.7 | 60.0 | 69.9 |
| **H** | 38.1 | 42.9 | 0.0 | *23.1* |
| **I** | 38.1 | 85.7 | 60.0 | 69.4 |
| **J** | 90.5 | 85.7 | 60.0 | 74.6 |
| **K** | 90.5 | 64.3 | 60.0 | 65.0 |
| **L** | 85.7 | 78.6 | 40.0 | 61.9 |
| **M** | 33.3 | 92.9 | 100.0 | 90.1 |
| **N** | 85.7 | 71.4 | 60.0 | 67.7 |
| **O** | 95.2 | 78.6 | 60.0 | 71.9 |
| **P** | 85.7 | 71.4 | 60.0 | 67.7 |
| **Q** | 71.4 | 64.3 | 60.0 | 63.1 |
| **R** | 42.9 | 71.4 | 100.0 | 81.4 |
| **S** | 95.2 | 71.4 | 100.0 | 86.7 |
| **T** | 90.5 | 71.4 | 100.0 | 86.2 |

**Scoring Details**

**Facilities**: Managers were asked to identify the sites in their area where a group education and exercise class for at least 6 – 8 people could be run. Then for each site, they were asked to assess the feasibility of providing the intervention. Current access to the site, funding secured for the site, and current use of the site for groups were prioritised and coded ‘2’ if present and ‘0’ if absent. Questions relating to the amenities available and accessibility of the site were coded ‘1’ if present and ‘0’ if absent. Facility scores were summed (maximum 14) and given a weight of 45% of the total score.

**Equipment:** For each identified site, Managers were asked whether they had the following equipment required to deliver the intervention prototype: therabands of varying resistance, floor mats, exercise balls, chairs, plinths, steps (stepper machine, box step), bouncer (mini trampoline), wedges or rollers to support knee raises, ankle weights or strap weights, stationary bikes (upright or recumbent), wall bars, parallel bars, motomed or pedals, pillows or cushions, towels, flip chart stand or audiovisual equipment. The minimum essential equipment (eg. therabands, floor mats, chairs and plinths) was prioritised and coded ‘2’ if present and ‘0’ if absent. All other equipment was scored ‘1’ if present and ‘0’ if absent. The equipment score was summed (maximum score 21) and given a weight of 10% of the total score.

**Staffing**: Managers were asked to identify specific physiotherapists who would be in a position to provide the intervention prototype, a group exercise and education programme, in each of the identified sites and to provide their grade, employment status and days/week dedicated to MSK. To assess the feasibility of the proposed programme delivery models (eg. one physiotherapist and one support staff) in each of the sites, managers were asked whether they currently had student placements or whether other health professionals were present at each of the sites. Lastly, Managers were asked whether they were expecting new staff, how many, when and whether these new staff would be involved in providing the study intervention. Sites were coded as follows: 5 - if they currently had two physiotherapists at the site (at least one of which was senior grade), 4 – if they currently had one senior grade at the site and were expecting additional staff to be in post in advance of the trial who would be involved in the study, 3 – if they currently had one senior grade (or two staff grade) at the site and students or other health professionals on site, 2 – if they currently had one senior grade at the site and no students or other health professionals, 1 – if there was one unsupported staff grade on site and 0 if there were no designated physiotherapists identified for the specified site.
