# Supplementary material for: Using intervention mapping to develop a theory-driven, group-based complex intervention to support self-management of osteoarthritis and low back pain (SOLAS)
Source: Implement Sci. 2016 Apr 26;11:56. doi: 10.1186/s13012-016-0418-2 (PMC4845501; doi:10.1186/s13012-016-0418-2)
Supplement: Supplementary file 4 — Overview of needs assessment results [112–114]. (DOCX 30 kb) [file 13012_2016_418_MOESM4_ESM.docx]

**Additional file 4 Overview of needs assessment results**

| **Methods employed** | **Contribution to evaluation of the health problem** |
| --- | --- |
| **Local service evidence** |  |
| Health service context  *National policy*  Transformation of health services for clients with chronic musculoskeletal pain from secondary acute hospital to primary care settings since 2011 [16]  Self-management framework for chronic disease management in public health services in Ireland  *Local Primary, Community and Continuing Care (PCCC) physiotherapy services Dublin, Kildare, Wicklow region*  Within this region, services are organised under nine local health offices: Dublin North, Dublin North Central, Dublin South City, Dublin South East, Dublin South West, Dublin West, Dun Laoghaire, Wicklow and Kildare/West Wicklow and domiciliary and clinic-based services are provided through a number of local primary care centres. | Increased demand for primary care services due to ageing population and rising numbers availing of publicly-funded health care leading to need to rationalise existing resources [112]  Increased demand on physiotherapy service to develop and deliver group-based programmes that promote self-management for clients with chronic diseases, including chronic musculoskeletal pain to increase efficiencies.  Physiotherapy managers (n=10) of all nine local health offices identified and contacted to explore health problem of delivering physiotherapy services for this population. |
| **Individual semi-structured interviews with physiotherapy managers and patients**  Each physiotherapy manager in Dublin, Kildare and Wicklow (n=10) completed a brief service description questionnaire prior to interview to provide context. Individual face to face semi-structured interviews were then conducted with the project manager (LCM) and principal investigator (DH).  Six patients with CLBP and/or spinal OA who had participated in a 6 week group physiotherapy education and exercise programme in the catchment area of the proposed feasibility trial were interviewed via telephone by a PhD student (ET).  All qualitative data were audio-recorded, transcribed verbatim, checked for accuracy, coded and analysed using deductive thematic analysis based on Braun and Clarke’s method using the framework approach [113], [114] based on the Theoretical Domains Framework (TDF) [39], [44]. In order to determine the reliability of the themes identified, a 20% sample of all interview data selected at random independently analysed by an independent rater with 70% agreement was taken as the minimum cut-off [52].  Additional file 1 provides details of the specific interview guides and coding frame. | Theoretical Domains Framework Domains [39], [44]  Manager Interviews  **Environmental context and resources**   - High level of demand and waiting lists for primary care physiotherapy services for patients chronic musculoskeletal pain, mainly osteoarthritis and chronic LBP requiring self-management support as part of treatment programme - Importance of multidisciplinary team but limited availability of psychologists, and dieticians to support development and delivery of self-management programmes - Variable access to suitable facilities and equipment   **Skills**   - Good expertise and experience in setting up group programmes for a range of other chronic conditions - Majority of staff currently managing patients with chronic musculoskeletal pain on an individual basis   **Intentions**   - Strong motivation to support staff to set up and deliver group programmes that promote self-management for this client group - Enthusiastic about collaborating with research team to support evaluation of feasibility of a group SM programme   **Beliefs about consequences**   - Reduced workload for staff through increasing efficient of caseload management and increased morale/job satisfaction - Social and peer learning benefits in supporting patients to self-manage   Patient interviews  **Social influences**   - Patients were positive about the social interaction they experienced as part of local group physiotherapy programme with other people experiencing the same condition from their area   **Beliefs about consequences**   - Positive outcomes in pain, mobility and ability to cope with their chronic low back pain on completion of the programme   **Environmental context and resources**   - Would have preferred a programme longer than 6 weeks to further support their ability to self-manage their condition   **Knowledge, skills, beliefs about capabilities**   - Increased understanding of their CLBP condition, self-management skills (physical activity, healthy eating, pacing) and confidence (self efficacy) in their use to manage their condition   **Optimism**   - Felt positive about the benefits they would receive from a future similar programme |
| **Literature** |  |
| Definition of self-management intervention [12] | A systematic, structured and multi-component intervention for people with chronic musculoskeletal pain that involves:  a) Education to increase knowledge about the individual’s chronic condition, its consequences, and its management, including the role of a healthy lifestyle and available community resources;  b) Training to impart skills, such as exercise and physical activity, pain coping skills, problem solving, decision making, communication, self-tailoring, goal setting, and action planning, and enabling participants to deploy enhanced skills in their lives beyond the intervention; with the aim of promoting adherence to SM behaviours, such as: i) self-regulation (including self-monitoring and effecting appropriate behavioural and emotional responses); ii) maintaining or increasing levels of activity, and iii) managing pain symptoms using coping strategies [12].  The ultimate goal of these interventions is to achieve improved physical (reduced functional disability, reduced pain, and decreased disease severity), psychological (improved well-being, improved psychological functioning, and increased quality of life), and economic (improved cost-effectiveness, increased satisfaction with service, return to work) outcomes. |
| Rapid review of effectiveness of group-based physiotherapist delivered education and exercise programmes that promote self-management  [inception-November 2013] [12] | 25 articles of 22 studies (n=10 OA, n=12 CLBP) of moderate to low risk of bias  No difference in the effectiveness of physiotherapist-led group education and exercise interventions that promoted SM compared to individual physiotherapy or medical management for people with OA or CLBP  No selected intervention included participants with OA and CLBP.  FASA intervention identified from literature review as potential intervention prototype for adaptation [35] |
| Clinical guideline recommendations for OA and chronic LBP re self-management behaviours  See additional file 5 for more specific details of the review | Osteoarthritis – individualised self-management strategies, positive behavioural strategies: regular exercise programme, physical activity, weight loss of overweight or obese, use of suitable footwear, pacing of activity [4]-[6]  Chronic low back pain – engage in physical activity, continuation or resumption of normal activities including work, use of activity pain coping strategies that reduce fear and catastrophizing [7], [47] |
| Review of systematic reviews of mediators of outcomes of self-management interventions in OA and chronic LBP | *Determinants of SM behaviour identified  Self-efficacy for OA and CLBP [48], [49]  Pain catastrophizing [48], [50]  Fear [51] |
| **Literature review of behaviour change theory and techniques in self-management programmes**  Any type of self-management programme delivered by healthcare professionals or lay leaders that support self-management for chronic musculoskeletal pain conditions predominantly OA and chronic LBP, as well as rheumatoid arthritis or fibromyalgia [75].  Group-based physiotherapist delivered education and exercise programmes that promote self-management in people with OA and chronic LBP [40] | Of 19 randomised controlled trials, 14 studies were based on theory frameworks; 11 studies adopted social cognitive theory, and 3 were based on cognitive behavioural theory.  2 of 22 randomised controlled trials, 2 studies were based on theory, both used social cognitive theory]  33 BCTs were coded, representing 35.4% of 93-item Behaviour Change Taxonomy v1 [43]  Most common BCTs in existing programmes identified  ‘instruction on how to perform the behaviour’ (100%, n =25)  ‘demonstration of the behaviour’ (100%, n= 25)  ‘behavioural practice’ (100%, n =25)  ‘credible source’ (80% n= 20)  ‘body changes’ (60%, n= 15)  ‘graded tasks’ (56%, n= 14)  Importance of underpinning planned intervention with theory was confirmed |
| Review of behaviour change theories [41] | Self-determination theory (SDT) [55] selected to underpin the adapted intervention  The theory proposes that autonomous motivation (and increased feelings of perceived competence) leads to greater persistence with the targeted behaviour, whereas controlled motivation (and lower feelings of perceived competence) can result in poor long term engagement with the targeted behaviour. According to SDT, autonomous motivation is characterised by self-endorsement of the behaviour and a belief in its value while controlled motivation typically relates to engaging in a behaviour due to feelings of guilt or external pressures. The development of autonomous motivation can occur through the social environment and the needs supportive behaviour of significant others, such as a health care practitioner.  *Determinants of SM behaviour – Motivation, behavioural regulation and perceived competence |
| **Focus groups** (n=2) with 28 Physiotherapists who  a) were currently providing group classes  b) had previously provided group classes  c) were likely to be providing group classes for people with CMP  Co-facilitated by an experienced qualitative researcher (SG), and the project manager (LCM). Principal investigator (DH) delivered series of brief presentations followed by discussion using a semi-structured topic guide to elicit physiotherapist attitudes towards the FASA intervention prototype and barriers and enablers to its implementation and participant recruitment based on the TDF. The interview guide and coding frame is provided in Additional file 1.  All qualitative data were audio-recorded, transcribed verbatim, checked for accuracy, coded and analysed. Deductive thematic analysis based on Braun and Clarke’s (2006) method [38] was conducted on the data using two coding frames in order to determine the feasibility of implementing the adapted FASA intervention in an Irish primary care context, as well as to identify potential barriers and enablers to its implementation respectively [113], [114]. A 20% sample of all interview data were selected at random and independently analysed by an independent rater (JM) with 70% agreement taken as the minimum cut-off to establish inter-rater reliability [52].  The first coding frame related to physiotherapists’ past experiences of and views on their acceptability of specific components of the proposed intervention. Data were coded to identify which key aspect it referred to and whether the attitude was positive (indicating acceptability) or negative (indicating unacceptability). Comments were then grouped according to the aspect that they addressed and analysed to identify key themes.  The second coding approach was based on the TDF [39] whereby the data were mapped directly onto the TDF domains, then further categorised under specific themes within each domain and then further coded as barriers or enablers to physiotherapists delivering the intervention prototype or recruiting participants to the trial. | Physiotherapist perspective  *Barriers to delivering intervention prototype*  **Theoretical Domains Framework**  **Environmental context and resources**   - securing and retaining sufficient clients - availability of suitable facilities and equipment to deliver the intervention as designed safely - increase in workload due to the time involved in setting up and running intervention   **Beliefs about their capabilities** to deliver the intervention as intended   - dealing with a mixed group of clients with varying physical abilities, motivation levels or joint conditions - communicating the education component on Pain effectively within a group format   **Beliefs about the consequences**   - causing flare-ups due to clients’ inability to appropriately select exercises - clients inability to self-regulate their activity levels during the intervention   *Enablers to delivering intervention prototype*  **Knowledge and skills**   - Significant experience in managing the target client groups - Experience in running group classes for a variety of chronic conditions   **Intentions**   - to set up group SM programmes within their health setting or local external organisation [i.e. gym, community centre]   **Optimism**   - about the SM approach and supporting client autonomy to self-manage   **Environmental context and resources**   - access to excellent facilities and equipment - possible input from other disciplines to support delivery in some areas   Participant perspective  *Barriers to engaging in intervention according to Physiotherapists*  **Knowledge and skills** in SM behaviours   - poor ability to engage in physical activity and exercise, pacing, and self-regulation   Low **motivation** to self-manage and regulate behaviour  Negative **emotions** about participating in a group, and undertaking exercise  *Enablers to engaging in intervention according to Physiotherapists*  **Social influences**   - Social support of group, promoting enjoyment, variety and interest [mix of people with OA and chronic LBP, varying motivation levels, education and exercise structure] - Importance of promoting sustainable lifestyle change through building on support opportunities from external local organisations (i.e. gyms) in promoting long-term self-management behaviours beyond the intervention.   **Goals**   - Use of within programme goals to support adherence and behavioural regulation   **Reinforcement**   - Provision of incentives (such as reduced gym membership) on successful completion of the programme to support long-term adherence.   *Determinants of client SM behaviour – Knowledge, Skills, Motivation, Behaviour regulation, Fear  **Summary of adaptations needed to FASA prototype to enhance feasibility**   - Participants [recruitment and detailed screening process, group size 6-8, mix of OA and CLBP, age <50yrs] - Content [education>15mins] - Structure [6 weeks, once weekly, >60mins] - Delivery [One physiotherapist delivering per site]   For further details see additional file 6. |
| **Resource capacity checklist**  Physiotherapy managers (n=10) completed detailed checklist of existing facilities, equipment and human resources within each potential clinic area (n=20) to identify potential study sites.  Score ≥80%: considered to have the requisite resources to provide the intervention as intended  Score 60– 80%: may require some tailoring of the intervention to the local context and resources  Score ≤60%: considered unable to provide the intervention as intended given current resource constraints. | Identification of 19 potential PCCC sites for the feasibility trial  n=10 (50%) scored ≥80%  n=9 (45%) scored 60 – 80%  n=1 (5%) scored ≤60% |
