# Supplementary material for: Using intervention mapping to develop a theory-driven, group-based complex intervention to support self-management of osteoarthritis and low back pain (SOLAS)
Source: Implement Sci. 2016 Apr 26;11:56. doi: 10.1186/s13012-016-0418-2 (PMC4845501; doi:10.1186/s13012-016-0418-2)
Supplement: Supplementary file 5 — Comparison of clinical guidelines for osteoarthritis and chronic low back pain to intervention prototype and adapted intervention [115–121]. (DOCX 37 kb) [file 13012_2016_418_MOESM5_ESM.docx]

**Additional file 5 Comparison of clinical guidelines for osteoarthritis and chronic low back pain to intervention prototype and adapted intervention**

Search strategy: 1. UK National Institute of Health and Care Excellence database searched for most recent guidance on osteoarthritis and low back pain. 2.‘clinical guideline’ search term was combined with ‘hip osteoarthritis’, ‘knee osteoarthritis’ or ‘low back pain’ search term, publication lists of Medline database searched for most recent guideline for each condition from international expert groups in OA and LBP.

| **Osteoarthritis Guidelines [4]-[6]** | **Chronic Low Back Pain Guidelines [7], [47]** | **FASA Intervention [35]**  **[intervention prototype]** | **SOLAS Intervention**  **[adapted intervention]** |
| --- | --- | --- | --- |
| **Education and Information** | |  |  |
| - Offer accurate verbal and written information to all people with osteoarthritis to enhance understanding of the condition and its management, and to counter misconceptions, such as that it inevitably progresses and cannot be treated. [6] - Ensure that information sharing is an ongoing, integral part of the management plan rather than a single event at time of presentation. [6] - All people with knee/hip OA should receive an individualised management plan that includes information and education regarding OA. [4] - To be effective, information and education should be individualised according to the person’s illness perceptions and educational capacity, specifically address the nature of OA (a repair process triggered by a range of insults), its causes (especially those pertaining to the individual), its consequences and prognosis, be reinforced and developed at subsequent clinical encounters, be supported by written/ and other types of information (eg DVD, website, group classes) and include partners or carers if appropriate [4] | - Provide people with advice and information to promote self-management of their low back pain - Offer educational advice that includes information on - the nature of non-specific low back pain - encourages the person to be physically active and continue with normal activities. [7]      - Clinicians should not utilize patient education and counselling strategies that either directly or indirectly increase perceived threat or fear associated with low back pain, such as strategies that (i) promote bed rest, (ii) provide in-depth pathoanatomical explanations for specific cause of patient’s low back pain, - Clinicians should emphasise - promotion of understanding of the anatomical/structural strength inherent in the human spine - neuroscience that explains pain perception - overall favourable prognosis of low back pain - use of active pain coping strategies that decrease fear and catastrophizing - early resumption of normal or vocational activities - importance of improvement in activity levels not just pain [47] | **Osteoarthritis**   - Accurate verbal and written information relevant to UK prevalence of osteoarthritis, factors influencing joint changes, pathophysiology of joint changes provided to enhance understanding and counter misconceptions [i.e. degenerative process, link between diet and joint pain on an ongoing basis during programme.   **Chronic low back pain**   - Not discussed specifically - Active coping strategies embedded within programme, including positive thinking, activity pacing, graded exposure, progressive goal setting, problem solving, promotion of physical activity and progressive muscle relaxation. | **Osteoarthritis**  Additional   - accurate verbal and written information related to prevalence of osteoarthritis in Ireland,   Removal   - information related to UK prevalence,   **Chronic low back pain**  Additional   - accurate verbal and written information on nature of non-specific chronic low back pain, - more detailed information on neuroscience explaining pain perception including pain pathways [115], - promotion of understanding of the anatomical/structural strength of the human spine, importance of good posture and safe lifting, - highlight favourable prognosis, - importance and rationale for early resumption of normal activities, including work if relevant,   Additional   - written and verbal education and discussion of active pain coping strategies to decrease fear and catastrophizing, - more education on rationale for staying active and avoiding rest [116], activity pacing and graded exposure techniques [115], - explanation of fear avoidance behaviour, and use of a biopsychosocial model that aims to overcome fear and avoidance of movement, - principles of cognitive behavioural management incorporated using an operant conditioning approach that aims to change participants’ behaviour by incorporating exercise and physical activity into their daily routine, reinforced by Physiotherapist - modification of attitude to pain, i.e. ‘hurt’ does not mean harm using cognitive restructuring, - provision of relaxation skills CD to encourage practice of progressive muscle relaxation. |
| **Patient self-management strategies** | |  |  |
| - Agree individualised self-management strategies with the person with osteoarthritis [6] - Ensure that positive behavioural changes, such as exercise, weight loss, use of suitable footwear and pacing, are appropriately targeted [6] - Ensure that self-management programmes for people with osteoarthritis, either individually or in groups, emphasise the recommended core treatments especially exercise [6] - All people with knee/hip OA should receive an individualised management plan that includes the core non-pharmacological approaches, information and education regarding OA, addressing maintenance and pacing of activity, addressing a regular individualised exercise regimen, addressing weight loss if overweight or obese, reduction of adverse mechanical factors (appropriate footwear), consideration of walking aids and assistive technology [4] - Self-management and education recommended for knee OA [5] | - Provide people with advice and information to promote self-management of their low back pain [7] - Offer educational advice that includes information on the nature of non-specific low back pain and encourages the person to be physically active and continue with normal activities [7] | **Osteoarthritis**   - Individual self-management strategies related to exercise and physical activity, appropriate evidence-based pain management, coping, healthy diet, and pacing are targeted but not measured. | **Osteoarthritis & Chronic Low Back Pain**  Additional   - self-management strategy related to weight management   Additional   - weekly review of progress in achieving self-management strategies by trained physiotherapist   Additional   - measurement of participants’ use of SOLAS self-management behaviours through new self-report measure developed for this research. |
| **Thermotherapy**   - The use of local heat or cold should be considered as an adjunct to core treatments [6] | - Not included in Clinical Guidelines [7],[47] | **Osteoarthritis**   - Information and education about safe application of heat and cold provided | **Osteoarthritis**   - No change   **Chronic low back pain**  Additional   - information and education about safe application of heat and cold provided to participants for pain self-management |
| **Exercise and physical activity** |  |  |  |
| - Advise people with osteoarthritis to exercise as a core treatment , irrespective of age, comorbidity, pain severity or disability. [6] - Exercise should include: local muscle strengthening and general aerobic fitness. It has not been specified whether exercise should be provided by the NHS or whether the healthcare professional should provide advice and encouragement to the person to obtain and carry out the intervention themselves. - Exercise has been found to be beneficial but the clinician needs to make a judgement in each case on how to effectively ensure participation. This will depend upon the person's individual needs, circumstances and self-motivation, and the availability of local facilities [6] - People with hip and/or knee OA should be taught a regular individualised (daily) exercise regimen that includes: strengthening exercises for both legs, aerobic activity and exercise, adjunctive range of movement/stretching exercises.[4] - Although initial instruction is required, the aim is for people to undertake these regularly on their own in their own environment [4] - Mode of delivery of exercise education (individual, group) and use of pools or other facilities should be selected according to preference of the patient and local availability. [4] - Important principles of all exercise include pacing, linking exercise to other daily activities so that they become part of lifestyle rather than additional events, starting with levels of exercise within individual’s capacity, but building up the dose sensibly over several months [4] - Exercise (land-based and/or water-based) and strength training recommended for knee OA [5] | - Advise people with low back pain that staying physically active is likely to be beneficial. Advise people with low back pain to exercise. [47] - Consider offering a structured exercise programme tailored to the person that should comprise up to a maximum of 8 sessions over a period of up to 12 weeks offered as a group supervised exercise programme in a group of up to 10 people. [7] - A one-to-one supervised exercise programme may be offered if a group programme is not suitable for a particular person [7] - Exercise programmes may include aerobic activity, movement instruction, muscle strengthening, postural control, stretching [7] - Clinicians should consider moderate to high intensity exercise for patients with chronic low back pain without generalized pain and incorporating progressive low-intensity, submaximal fitness and endurance activities into the pain management and health promotion strategies for patients with chronic low back pain with generalized pain [47] | **Osteoarthritis**   - Benefits of exercise [physical, psychological, social], physical activity recommendations [30 minutes, moderate intensity most days, short bouts or in one go], given and discussed. - Supervised group exercise programme twice per week x 6 wks - *Local muscle strengthening* exercises hip abductors, extensors, flexors, knee extensors, back extensors, abdominals - *Flexibility exercises* hip flexion, extension, adduction, knee flexion, extension, lumbar spine flexion, extension, rotation. - *General aerobic exercises*: shuttle walking, treadmill [if available], bouncer, stationery cycling, step ups, sit to stand, wall slides, wall press ups [75] - Provision of exercise leaflet with written instructions and photographs of young male with no pain doing each exercise - Weekly goal setting and action planning using activity diary | **Osteoarthritis and Chronic Low Back Pain**  Additional   - *Get Ireland Active* physical activity recommendations which are consistent with international recommendations [i.e. people with chronic conditions/over 65 years: at least 30 minutes moderate intensity activity, or as much as ability allows, 5 days a week, short bouts of at least 10 minutes can be accumulated [117, 118]   Additional   - written and verbal information and group discussion about benefits of walking and good walking technique [119]   Additional   - pedometer as self-monitoring tool provided to each participant, - written and verbal explanation and demonstration of how to use a pedometer during group education session [117]   Additional   - adapted weekly activity diary to incorporate step count,   Additional   - provision of written information about local opportunities to be physically active tailored to each participating clinic [117]   **Osteoarthritis and Chronic Low Back Pain**  Supervised group exercise programme up to 8 people once per week x 6 wks  Additional   - postural control [controlled movement sitting on gym ball], - muscle strengthening abdominals   Additional   - participants advised to conduct home exercise programme at least twice per week [88]   Additional   - provision of exercise theraband if available   Additional   - production of new exercise leaflet with written instructions and photographs of a male aged 60 years with osteoarthritis of both knees and back pain doing exercises   Adaptation   - weekly goal setting and action planning using activity diary to include step count |
| **Weight loss**   - Offer interventions to achieve weight loss as a core treatment for people who are obese or overweight [6] - Education on weight loss should incorporate individualised strategies that are recognised to effect successful weight loss and maintenance for example: - regular self-monitoring, - recording monthly weight, - regular support meetings to review/discuss progress - increase physical activity, - follow a structured meal plan that starts with breakfast, reduce fat and sugar, limit salt, increase intake of fruit and vegetables (at least 5 per day). - limit portion size, - address eating behaviours and triggers to eating - nutrition education, - relapse prediction and management (eg alternative coping strategies) [4] - Weight management recommended for knee OA [5] | - Not included in current guidelines [7], [47] | **Osteoarthritis**   - Healthy diet and importance of keeping weight at an appropriate level. - No specific information given to participants about healthy weight targets, or how to measure it, or weight loss if relevant. | **Osteoarthritis and Chronic Low Back Pain**  Additional   - education on healthy eating and balanced weight as detailed below: - written and verbal education communicating the benefits of maintaining a healthy weight tailored for this specific population [120], - statistics from current Irish research on prevalence of overweight/obesity related to age/gender, life expectancy, and disability due to chronic pain [3] - education on portion sizes [120] - encouragement of dietary habits that reduce the risk of excess energy intake [120] by provision of Healthy Eating guide* and 101+ square meals cookbook^#^ - issue of [weight loss](http://www.nice.org.uk/guidance/ph53/chapter/glossary#weight-loss) raised in a respectful and non-judgemental way, while recognising that this may have been raised on numerous occasions and respecting someone's choice not to discuss it further on this occasion [121] - written and verbal education about self-monitoring of waist size, risk level and gender, - demonstration of measuring waist size - provision of a tape measure for home use [120], [121]   Additional   - promotion of physical activity and walking in particular to avoid low energy expenditure [see Exercise above] [120]   Additional   - referral of overweight or obese participants to a lifestyle weight management programme if appropriate and willing [121] |
| **Electrotherapy**   - Healthcare professionals should consider the use of transcutaneous electrical nerve stimulation (TENS) as an adjunct to core treatments for pain relief [6] - Uncertain if TENS should be recommended for knee OA [5] | - Do not offer TENS [7] | **Osteoarthritis**   - Education about TENS effects and safety precautions and where units can be purchased | **Osteoarthritis**  Additional   - information on health service loan schemes and cost of purchasing TENS unit   **Chronic low back pain**   - information provided to participants who may choose to use TENS for pain self-management |
| **Nutraceuticals**   - Do not offer glucosamine or chondroitin products for the management of osteoarthritis. [6] - Glucosamine for symptom relief [uncertain recommendation] and for disease modification not appropriate for knee OA [3] | - Not included [7],[47] | **Osteoarthritis**   - Information about glucosamine and chondroitin dosage and where to purchase. Conflicting evidence highlighted. | **Osteoarthritis**  Removal   - information about glucosamine or chondroitin is provided. |
| **Acupuncture**   - Do not offer acupuncture for the management of osteoarthritis [6] - Acupuncture uncertain recommendation for knee OA [5] | - Consider offering a course of acupuncture needling comprising up to a maximum of 10 sessions over a period of up to 12 weeks [7] | **Osteoarthritis**   - Education stating no supportive evidence in arthritis but it may offer short-term pain relief. | **Osteoarthritis**   - education changed to state acupuncture not recommended for arthritis.   **Chronic low back pain**   - education that acupuncture is recommended for back pain, - information provided on how individual could access acupuncture in the health service if available locally. |

*Your Guide to Healthy Eating using the Food Pyramid was produced by the Health Service Executive and the Department of Health, Republic of Ireland. ^#^101+ Square Meals is a freely available book developed by Safefood Ireland that supports the implementation of Healthy Ireland (hi) the National Framework aimed at improving the health and wellbeing of everyone in Ireland. It supports every person to stay as healthy as possible throughout their life. The book uses the Irish Food Pyramid and Healthy Eating Guidelines to help readers plan healthier meals and get best value for money. It contains selected recipes and shopping tips for ingredients in season or on offer, food safety messages, menu planning advice, and treats and snacks for special occasions to support readers to have a varied and healthy diet on a budget.
