# Supplementary material for: Using intervention mapping to develop a theory-driven, group-based complex intervention to support self-management of osteoarthritis and low back pain (SOLAS)
Source: Implement Sci. 2016 Apr 26;11:56. doi: 10.1186/s13012-016-0418-2 (PMC4845501; doi:10.1186/s13012-016-0418-2)
Supplement: Supplementary file 6 — Results of feasibility analysis - focus groups. (DOCX 26 kb) [file 13012_2016_418_MOESM6_ESM.docx]

**Additional file 6 Results of feasibility analysis – focus groups**

| **Intervention Components** | | **Experience & Attitudes** | | **Factors requiring adaptation** |
| --- | --- | --- | --- | --- |
| 1. **Programme participants** | | **Negative** | **Positive** |  |
| **Number of suitable participants** | **Recruitment**  **Selecting most appropriate participants**  **Number of participants in a class** | **“***We tried to start a class and we couldn’t continue it because of lack of interest from the patients”*  **“***I cover two primary care teams and my colleague covers two primary care teams so we have four primary care teams to draw upon and we struggled to find the right people for our classes. Four huge vast areas, densely populated areas and we struggled.”*  **“***How do you select the right person, like what is the right person?”*  I’d be kind of concerned that it’s just throwing the net very wide I mean I just am, I could if it’s from referrals from within the service in general that it could be sort of a bit of a catch all maybe.”  *“it becomes very disheartening when you start a programme and a couple of people cry off or just stop showing up..it really becomes counterproductive then it’s a waste of your time..you mentioned 4 as a minimum, but i think for me I would need more than 4 in a class to justify running a class for two hours a week”*  *‘* | *“I think if it worked it would be great for your caseload in primary care because a huge chunk of our patients are chronic musculoskeletal”*  *“what the response has been for people, it’s been about 80% have been very open to the idea of a group even after having been seen maybe once or twice one to one so I don’t think that would be a big problem”*  *“ I think balancing ability would be a big thing because again a lot of patients would be elderly and I suppose you just have to have very strict inclusion criteria”*  *“they’d have to be screened in some way”*  *“I think its better to have eight people because otherwise I think the dynamic of the group would be, I think if there isn’t a group then it’s not a group”* | Maximise number of suitable referrals that can be included  Detailed screening process  Proposed class size 6-8 participants |
| Mixed group | Participants with OA and LBP | *“let’s say there’s eight people there and they are a mix of hips, knees and backs…its easy to say yes they all do their own exercises but in reality peolle aren’t going to remember what they exercises are doing..rather than it being a back class, a hip class or a knee class”*  “ *I just wonder about you know the mixing back pain wth knee and hip because a lot of the research is saying that back pain should be more, if you target the specific people with back pain and put them in the right classes and treat them the right way then that’s going to get them better”* | “*the knees and the hips or the knees and the backs there are, there’s a lot of things they can do together”.*  *“I think a positive thing about mixing the groups is that sometimes with back groups people can, use a lot of the time to talk about their pain in a negative way and sometimes it can turn into everyone complaining about their back pain together so I think the mix could be good from that point of view that ‘oh it’s your hip, it’s your knee, it’s your back’ so no one is getting too overly focused on the pain so that could be positive.”* | Include a mixed group of participants with OA and LBP |
|  | Participant age | “*when we did a general exercise class before..you try and get say the younger people to participate, they are so aware that there is an older generation there that generally they didn’t stick at it. They’re looking around kind of comparing things here”*  *“ No I think a lot of it is to do with age, you know somebody who is quite old and somebody who is quite young and they are put into the same group, I mean that can potentially drag down somebody who is quite young thinking why am I in with these old people”* | “*You could have maybe someone in their 20s and then someone in their 60s like a huge range. But it doesn’t really stop them, like circuit training and they all kind of know what they are doing themselves they don’t need too much direction and they are just happy to just get on with it. And in terms of their presenting complaint they are all very similar, they are able to share in their experiences despite their age differences… They enjoy being able to share their experiences despite their age difference, they are able to empathise with each other”* | Consider lowering age limit below 50 years |

| 1. **Programme Content** | |  |  |  |
| --- | --- | --- | --- | --- |
| Education | **Time allotted 15 mins** | **“** *there tends to be a lot of questions I find when we do our education talks, people have lot of questions and I think 15 minutes is for you just doing your but but does not allow for any kind of Q&A session for the people attending”*  *“Probably 15 minutes initially should be fine, but ... sometimes the discussions can go on longer and they nearly use it as a sort of support session. You can get one or two people who take over because they just want to vent about their feelings, so I suppose you very much have to kind of stick to the education and not really open it up to the floor too much for discussion if its only that 15 minutes’* | *‘15 minutes education and that’s the intervention, but sometimes you get a group where you’re getting a really good discussion, you might be getting more out of that then anything, so you don’t just want to cut it off straight away, so then maybe you give it half an hour’*  *“ it seems like a good split,people would stay interested, but beyond 15 wouldn’t be good because of people’s attention”* | Increase education time to allow more discussion |
| Exercise | **Circuit-based** | **“***I would wonder how you would progress on from the knee and the back..to keep them interested or to progress as they need to be progressed..is that depth of exercises included to keep them interested or going?”* | *“I think its good they can choose their own and what they feel like doing..”*  *“ I suppose the interesting thing is they can do it at their own pace.its great cause obviously its better for self-management”*  *“with circuit training they all kind of know what they are doing themselves they don’t need too much direction and they are just happy to get on with it”* | Continue with circuit-based format |
| 1. **Programme Structure** | |  |  |  |
| Group-based |  | *“I don’t have any experience with chronic musculoskeletal classes”*  *“ to be honest I don’t think these classes reduce the waiting list…because there are very few you can just take off the waiting list and send straight to a class”* | “*I think the experience of the physios would have a lot to do with it..if a physio hasn’t done a group setting before then they have eight new patients and a whole new challenge whereas if someone’s done that a few times before and theyre used to it, its less of a panic”*  *“I suppose you find with chronic patients that get referred back in so from a time efficiency point of view, groups when they work are better for you from a time management point of view!* | Continue with group-based format |
| 6 weeks | **Current practice**  **Rolling-programme**  **[ie clients can start at different weeks and the programme continues to run over an extended time period]** | *‘previously when we have done classes I thought 8 weeks was quite good. Not twice a week, once a week for eight weeks. You know you talk about this wheel of cognitive behaviour therapy trying to engrain something, ah three weeks they are just getting to know it, six weeks they are quite good, you are feeling by eight weeks they should be quite comfortable with it that it comes straight to them.’*  *“I’ve got a few people who once they get the six weeks you have them, you know well done, but it’s to get them there but I would have found over a six month period only 50% of the people who were referred to me made it to six weeks. And I feel if I can get 50% through the programme I am doing ok’* | *Six is kind of normal and would be what most people would do, I am at around that’*  *“we’ve run exercise classes on and off for about two or there years. They run for six weeks, originaly it was for longer but they run for six weeks now”*  *“if you missed a week because you were away or if you were sick you can just keep carry on to the next week.”*  *it’s not everyone starting on the same day so you might have half the group there and they have done maybe three sessions already they are fairly familiar with the setup and then three new people coming in’* | Continue with 6 week format  Present reasons for start-stop programme to physiotherapists |
| Twice weekly | **Ideal but not practical** | ‘*Twice a week is …. (pause, laughter from the group) … A nice idea. What you use in trials and then never use in practice’*  *‘Twice a week isn’t feasible and once a week is probably what we can manage’*  *‘…from the patients side as well you’re looking at people who have chronic conditions possibly don’t exercise and who have never exercised in their lives anyway and you’re trying to tell them to buy into a programme, it’s twice a week and that’s going to be quite difficult for them to start with’.* | *“it certainly sounds good in theory, I think it would be worth trying to set up something like that”*  *“We ran pulmonary rehab twice a week in the community but that was because that was based on the gold standard, which is twice a week for pulmonary rehab”* | Run programme once per week |
| 1-hour class | **Current practice** | *“At the minute we run it once a week, and we started with the one hour but I found we never could get it done if people were late so you know, now it’s an hour and a half almost.”* | “*it would be an hour or an hour and a half, there’d be a bit of flexibility depending on how long the discussion went on”* | Increase time of each class |
| 1. **Programme Delivery** | |  |  |  |
| Staffing | **One physiotherapist running the programme** | *“with one therapist going around and with everyone doing different exercises trying to explain them all I think you can come into problems there”.*  *“..for one physio to do it on their own, you know just for safety. I think it is nicer to have somebody there in case I suppose. Especially with our population you could have fallers”.* | *“To me it’s a lot more realistic to run a class with just one physio, ah where there isn’t, again I’m working by myself in ________, I’m reliant on someone from a different area coming in then, and if you had holidays then it becomes very disruptive then I think it’s a lot easier to run with one”*  *“I think if you have the option of having a physio assistant to help out in the classes, instead of two physios”*  *“Students have been excellent for us with the support they give us for our classes”* | Aim for consistency in staffing in relation to number of physiotherapists delivering programme across sites |
