# Supplementary material for: Using intervention mapping to develop a theory-driven, group-based complex intervention to support self-management of osteoarthritis and low back pain (SOLAS)
Source: Implement Sci. 2016 Apr 26;11:56. doi: 10.1186/s13012-016-0418-2 (PMC4845501; doi:10.1186/s13012-016-0418-2)
Supplement: Supplementary file 7 — Matrix of change objectives for self-management behaviour. (DOCX 19 kb) [file 13012_2016_418_MOESM7_ESM.docx]

**Additional file 7 Matrix of change objectives for self-management behaviour**

|  | **Determinants of Self-Management Behaviour from Needs Assessment linked to TDF Domains** | | | | | | |
| --- | --- | --- | --- | --- | --- | --- | --- |
|  | **Knowledge** | **Skills** | **Beliefs about capabilities** | **Beliefs about consequences** | **Intentions and goals** | **Behavioural regulation** | **Emotion** |
| PO.1  Accepts the benefits of physical activity | Develops an understanding of the benefits of physical activity |  |  |  | Increase autonomous motivation to engage in physical activities |  |  |
| PO.2 Selects PA (s) relevant to lifestyle/pain condition | Develops an understanding of recommended types and levels of physical activity |  | Improve self-efficacy in ability to perform selected physical activities | Reduce pain catastrophizing beliefs’ related to consequences of engaging in physical activity | Increase autonomous motivation to engage in selected physical activities |  |  |
| PO.3 Performs selected PA(s) | Develops an understanding of how to perform selected physical activity | Develops physical skills to engage in physical activity | Improve self-efficacy in ability to engage in selected physical activities | Reduce pain catastrophizing beliefs’ related to consequences of engaging in physical activity | Increase autonomous motivation to engage in selected physical activities | Develops ability to incorporate and monitor effects of physical activity into daily life | Reduces fear related to engaging in selected physical activities |
| PO.4 Uses SMART goal setting for the selected PA(s) | Develops an understanding of SMART goals and its relevance for physical activity | Develops skills to apply relevant SMART goal setting to selected physical activity | Improve self-efficacy in ability to use SMART goal setting |  | Increase autonomous motivation to use SMART goal setting |  |  |
| PO.5 Uses pacing to support selected PA (s) | Develops an understanding of pacing and its relevance for physical activity | Develops skills to apply pacing to selected physical activity | Improve confidence in ability to use pacing |  | Increase autonomous motivation to use pacing | Develops ability to incorporate and monitor effects of physical activity into daily life |  |
| PO.6 Monitors progress in increasing PA | Develop an understanding of tools for monitoring progress in PA | Develop skills in monitoring progress in increasing PA | Improve confidence in ability to use tools to monitor progress |  | Increase autonomous motivation to use tools to monitor progress in PA | Implement tools to monitor PA progress |  |
| PO.7 Copes with the challenges encountered with engaging in selected PA | Develop an understanding of typical challenges while engaging in PA | Develop skills to cope with the challenges encountered while engaging in selected PA | Improve confidence in ability to cope with challenges encountered during PA participation | Reduce pain catastrophizing beliefs’ related to consequences of engaging in physical activity | Increase autonomous motivation to cope with challenges encountered during PA participation |  | Reduce fear related to engaging in selected physical activities |
| PO.8 Identifies long-term PA plan | Develop understanding of available resources/facilities’ to support participation in PA |  | Improve confidence to engage in long-term PA | Reduce pain catastrophizing beliefs’ related to consequences of engaging in long-term physical activity | Improve autonomous motivation to engage in long-term PA |  | Reduce fear related to engaging in selected long-term physical activities |

|  | **Knowledge** | **Skills** | **Beliefs about capabilities** | **Beliefs about consequences** | **Intention and goals** | **Behavioural regulation** | **Emotion** |
| --- | --- | --- | --- | --- | --- | --- | --- |
| PO.9 Accepts the role of SM approach | Develops an understanding of the rationale for self-management |  |  |  | Increase autonomous motivation to self-manage their pain condition |  |  |
| PO.10 Selects appropriate evidence-based pain management strategies to self-manage pain condition | Develops an understanding of evidence-based pharmacological and non-pharmacological pain management strategies relevant to their pain condition | Develop skills to select and use evidence-based pharmacological and non-pharmacological pain management strategies relevant to their pain condition | Increase self-efficacy to use evidence-based pharmacological and non-pharmacological pain management strategies relevant to their pain condition | Reduce pain catastrophizing beliefs’ associated with pain condition by using pain coping strategies | Increase autonomous motivation to use evidence-based pharmacological and non-pharmacological pain management strategies relevant to their pain condition | Develop ability to monitor pain condition to select and apply evidence-based pharmacological and non-pharmacological pain management strategies relevant to their pain condition | Reduce fear associated with pain condition by using pain coping strategies |
| PO.11 Uses pain coping strategies | Develops an understanding of pain coping strategies | Develop skills to select and use pain coping strategies | Increase self-efficacy in ability to use coping strategies | Reduce pain catastrophizing beliefs’ associated with pain condition by using pain coping strategies | Increase autonomous motivation to use pain coping strategies | Develop ability to monitor pain condition to select and apply appropriate pain coping strategies | Reduce fear associated with pain condition by using pain coping strategies |
| PO.12 Applies healthy eating guidelines for healthy lifestyle and to support weight management if appropriate | Develops an understanding of healthy eating guidelines and healthy weight | Develop skills to follow healthy eating guidelines’ and monitor weight | Increase self-efficacy in ability to follow healthy eating guidelines and monitor healthy weight |  | Increase autonomous motivation to follow healthy eating guidelines and monitor healthy eating and weight | Develop ability to monitor healthy eating and weight |  |
| PO.13 Uses specific exercise for pain condition | Develops an understanding of how to perform selected specific exercises | Develops physical skills to engage in specific exercises | Improves self-efficacy in ability to engage in specific exercises | Reduce pain catastrophizing beliefs’ related to consequences of engaging in specific exercises | Increase autonomous motivation to engage in selected specific exercises | Develop ability to incorporate and monitor effects of specific exercise in daily life | Reduces fear related to engaging in selected specific exercises |
