# Supplementary material for: Using intervention mapping to develop a theory-driven, group-based complex intervention to support self-management of osteoarthritis and low back pain (SOLAS)
Source: Implement Sci. 2016 Apr 26;11:56. doi: 10.1186/s13012-016-0418-2 (PMC4845501; doi:10.1186/s13012-016-0418-2)
Supplement: Supplementary file 8 — Matrix of change objectives for adoption and implementation. (DOCX 18 kb) [file 13012_2016_418_MOESM8_ESM.docx]

**Additional file 8 Matrix of change objectives for adoption and implementation**

|  | **Determinants of Adoption and Implementation Behaviour from Needs Assessment linked to TDF Domains** | | | | | | |
| --- | --- | --- | --- | --- | --- | --- | --- |
| **Performance objective [PO]** | **Knowledge** | **Skills** | **Beliefs about capabilities** | **Beliefs about consequences** | **Environmental context and resources** | **Intentions** | **Social influences** |
| PO.1  Physiotherapy managers adopt the SOLAS intervention and participant recruitment procedures within their PCCC service area |  |  |  |  | Allocate resources to support service to deliver the SOLAS intervention | Provide written agreement to allow their service to participate in SOLAS feasibility trial |  |
| PO.2  Physiotherapists agree to participate in the SOLAS feasibility trial |  |  |  |  |  | Provide written agreement to participate in SOLAS feasibility trial |  |
| PO.3  Physiotherapists complete training in the content and delivery of the SOLAS intervention | Develops an understanding of the structure, content, materials, and needs supportive interpersonal delivery style within the SOLAS intervention | Develops skills in delivering the SOLAS intervention | Improve confidence in ability to deliver the SOLAS intervention | Reduce concerns about potential for clients to experience flare-ups during the SOLAS intervention |  |  |  |
| PO.4  Physiotherapists prepare local site to support delivery of SOLAS intervention |  |  |  |  | Improve physical environment to prepare for delivery of the SOLAS intervention |  | Adapt social environment to prepare for delivery of the SOLAS intervention |
| PO.5  Support participant recruitment to the SOLAS intervention within the feasibility trial | Develop GPs understanding of the participant recruitment pathway to the SOLAS intervention and feasibility trial |  |  |  |  |  | Increases awareness of the SOLAS intervention, feasibility trial and participants recruitment pathway to referring GPs, primary care team and potential participants |
